# The *Sinorhizobium meliloti* chemoreceptor McpU mediates positive and negative chemotaxis responses to dipeptides and competes with periplasmic solute-binding proteins for ligand binding

**DOI:** 10.1128/mbio.01739-26

**Published:** 2026-08-20

**Authors:** Foster K. Agyei, Nisha Dhiman, Birgit E. Scharf

**Affiliations:** 1 Department of Biological Sciences, Life Sciences I, Virginia Tech 1757, Blacksburg, Virginia, USA; Massachusetts Institute of Technology, Cambridge, Massachusetts, USA

**Keywords:** ABC transporters, *Medicago sativa*, *Rhizobium*, swimming velocity, transmembrane receptors

## Abstract

Many bacteria can utilize extracellular amino acids and peptides as nitrogen sources, metabolic energy, or as an indicator of the environment. *Sinorhizobium meliloti*, an endosymbiont of alfalfa, is attracted to host-exuded amino acids sensed by the chemoreceptor McpU, but the role of peptides in *S. meliloti* chemotaxis is unknown. A high-throughput screen using Biolog compound arrays confirmed efficient peptide utilization by *S. meliloti* with a preference toward nonpolar peptides. Differential scanning fluorimetry and isothermal titration calorimetry (ITC) demonstrated binding of non-aspartate dipeptides to the purified, periplasmic ligand-binding domain of McpU (McpU^LBD^), which was abolished by a single amino acid substitution in the McpU ligand-binding pocket. *S. meliloti* wild-type*, but not the mcpU*, deletion strain elicited positive chemotaxis to specific dipeptides in a quantitative capillary assay. However, chemotactic responses of *S. meliloti* wild type to the dipeptides Ala-Ala, Arg-Trp, and Phe-Ala were observed at 10- to 100-fold higher concentrations as compared to its response to the prototypical McpU ligands, proline and alanine, while cells did not migrate into capillaries filled with Trp-Ala and Val-Phe. We showed that this shift in chemotaxis response might be explained by dipeptides binding to solute-binding proteins, thereby decreasing the concentration of free dipeptides in the periplasm. *S. meliloti* regulates chemotaxis responses by controlling its swimming velocity. Consequently, a repellent response is expected to result in a reduction in swimming speed. Swimming speed measurements of wild type and a strain deficient in chemotaxis revealed negative chemotaxis of *S. meliloti* toward dipeptides. However, the response at higher concentrations of Trp-Ala was dominated by a detrimental effect on swimming motility. Agarose chemical-in-plug assay confirmed negative chemotaxis to dipeptides mediated by McpU. This study provides the first report of negative chemotaxis responses in *S. meliloti*.

**IMPORTANCE:**

Most gram-negative bacteria use small peptides as carbon sources and signaling molecules, which contributes to their survival in a wide range of environmental conditions. The ability to detect and navigate the environment for these peptides, along with the necessary systems for their metabolism, is crucial. *Sinorhizobium meliloti*, an endosymbiont of alfalfa, significantly enhances the growth of its host by fixing atmospheric nitrogen. This study provides firsthand information on peptide physiology and the possible role of dipeptide transporters in *S. meliloti*'s chemotaxis to dipeptides. Furthermore, we provide the first description of negative chemotaxis in *S. meliloti*. Specifically, the *S. meliloti* chemoreceptor McpU transmits positive and negative chemotactic responses to dipeptides. Understanding the mechanisms that enable *S. meliloti* to adapt to and survive in its environment could have a significant impact on the development of commercial bacterial inoculants, reducing our dependence on synthetic fertilizers that harm the environment.

## INTRODUCTION

Chemotaxis is a mechanism that allows flagellated bacteria to detect and move toward optimal environmental conditions that enhance their chances of survival (1, 2). The success of these chemotactic movements depends on accurately identifying environmental signals through methyl-accepting chemotaxis proteins (MCPs) or chemoreceptors that transduce these signals across the cell membrane to cause a cellular response (3). Canonical chemoreceptors are structured into an input and an output module connected by two transmembrane helices. Typically, the periplasmic input module consists of a single global domain; however, in certain chemoreceptors, it may be formed by two or more domains (4). The cytoplasmic output module is characterized by an extended dimeric four-helix bundle (4HB) consisting of two symmetric antiparallel coiled coils (5). The cytoplasmic signaling domain is functionally divided into the HAMP (histidine kinase, adenylyl cyclases, methyl-binding proteins, and phosphatases) domain, the methylation helix bundle, the glycine hinge (GH), and the kinase control module (6).

Bacterial chemoreceptors can sense a diverse array of stimuli, such as aromatic molecules, organic and inorganic compounds, pH, temperature, osmotic pressure, redox, and oxygen, which can elicit an attractant or repellent response (7–10). Attractant or repellent responses are further influenced by the chemoeffector’s concentration (11).

*Escherichia coli* has been the most extensively researched organism for understanding the underlying signaling mechanisms in response to a concentration gradient of chemical attractants or repellents (12). *E. coli* uses five chemoreceptors, namely, Tap, Tar, Trg, Tsr, and Aer, to move toward and away from various compounds (13). These chemoreceptors sense compounds through direct and indirect binding of chemoeffectors. For example, while Tar facilitates chemotaxis to aspartate through direct binding (14, 15), it also senses maltose by binding to a maltose-binding protein complexed with maltose (16). In *E. coli*, the chemotaxis signal transduction mechanism in response to attractant or repellent binding involves a dimeric histidine kinase, CheA. CheA is linked to the chemoreceptors through the protein CheW. Attractant binding causes a conformational change in the cytoplasmic HAMP domain of the chemoreceptor mediated by the transmembrane helices that strongly reduce the autophosphorylation of CheA. Conversely, autophosphorylation of CheA occurs at a high rate in the absence of a ligand or presence of a repellent. CheA subsequently phosphorylates the response regulator CheY at a conserved aspartate residue. Phosphorylated CheY directly interacts with the flagellar motor complex, which causes a directional change of rotation from counterclockwise to clockwise resulting in tumbling (2, 13, 17, 18). An adaptation system characterized by reversible methylation and demethylation of chemoreceptors at specific modification sites catalyzed by the methyltransferase CheR and the methylesterase CheB~*P* is employed to sustain baseline activity and enhance sensitivity to ligands.

*Sinorhizobium meliloti* is a gram-negative, free-living soil bacterium, which is also capable of existing as an endosymbiont of *Medicago sativa* (alfalfa) (19). The ability of *S. meliloti* to establish a nitrogen-fixing symbiotic relationship with alfalfa ensures enhanced growth of this crop plant in an arable environment, simultaneously reducing reliance on expensive and environmentally detrimental synthetic nitrogenous fertilizers (20). *S. meliloti* uses flagellar motility to actively swim in the soil and colonize the roots of alfalfa plants in response to chemical stimuli. Eight chemoreceptors in *S. meliloti* mediate the chemotaxis response that precedes symbiosis and initiates the beneficial association between the two partners. We demonstrated that McpU is the primary chemoreceptor for recognizing amino acids, which are one major attractant group in host exudates (21, 22). Additionally, we showed that McpT, McpX, and McpV sense a broad range of carboxylates, quaternary ammonium compounds, and short-chain carboxylates, respectively (23–25). However, the ligand spectrum of the remaining receptors is unknown.

Finding favorable, nutrient-rich rhizosphere niches is essential for the survival of soil bacteria due to rapidly changing environmental conditions (26). Their unique ability to direct movement within concentration gradients of chemoattractants and repellents significantly enhances persistence and colonization of plant roots (27, 28). Bacteria use an array of transport systems for essential nutrients to foster optimal growth in an extremely competitive environment. Periplasmic solute-binding proteins (SBPs) serve as molecular shuttles that provide substrates to membrane transporters. Rhizosphere bacteria, such as *S. meliloti*, *Mesorhizobium loti*, and *Agrobacterium tumefaciens*, possess an unusually high number of SBPs (29–32). The large quantity of SBPs may have led to an evolutionary advantage of Rhizobia in the acquisition of a variety of growth-restricting nutrients from the soil and rhizosphere for their survival and fitness (33). A variety of small molecules in the soil, such as peptides, can serve as a direct source of nitrogen and provide energy for soil microbes (34). Gaining access to nitrogen sources from decomposing biological materials, including amino acids and peptides, would allow organisms to outcompete those that depend only on inorganic sources, thereby circumventing the nitrogen cycle (32). However, it is unknown whether Rhizobia recognize peptides as chemoattractants, and information about the physiology of peptide metabolism, other than the analysis of three ABC transporters for oligopeptides, is lacking (35). Here, we have explored peptide metabolism and chemotaxis in *S. meliloti* and discovered an unexpected relationship between both bacterial traits.

## RESULTS

### *S. meliloti* uses peptides as a nitrogen source

**Fig 1:**
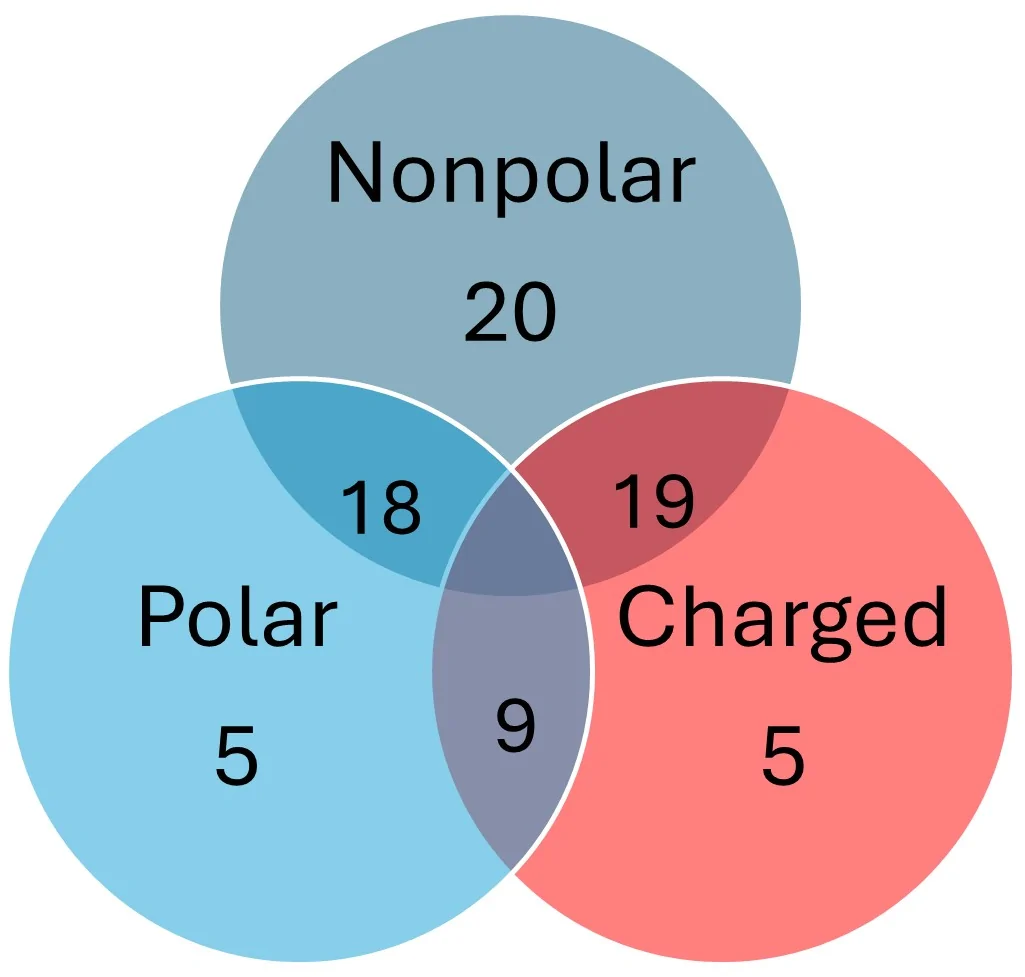
Classification of 76 peptides efficiently utilized by *S. meliloti* as analyzed in the Biolog growth assay. Peptide classification revealed the preference of *S. meliloti* for peptides based on constituent polar or non-polar amino acids with a dominance for peptides with at least one non-polar amino acid.

To assess the peptide utilization profile of *S. meliloti*, we employed Biolog phenotypic microarray plates PM6 to PM8 containing peptides as nitrogen sources. These plates included 282 different peptide compounds, of which 268 were dipeptides, and 14 were tripeptides. This enabled the thorough evaluation of a variety of peptides *S. meliloti* RU11/001 can utilize by reducing the tetrazolium dye present in the plates to the purple-colored formazan, which allowed the direct correlation of cellular respiration through absorbance at 590 nm (36). Peptides in wells with an absorbance ≥0.2 were considered to promote metabolic activity, whereas those with an absorbance ≥0.4 were considered to promote efficient utilization. The initial data analysis revealed that *S. meliloti* was able to metabolize 240 peptides as a nitrogen source, which included 228 dipeptides and 12 tripeptides. A full list of the utilization profile of all peptides is found in Tables S1 to S3. Further analysis allowed the conclusion that 76 peptides were efficiently utilized by *S. meliloti* (well absorbance ≥ 0.4). These 76 peptides could be classified into polar-polar, polar-nonpolar, and nonpolar-nonpolar amino acids based on their constituent units. This classification further revealed the dominance of nonpolar peptides as substrates of preference by *S. meliloti* (Fig. 1). These included 57 peptides constituting either non-polar amino acids or at least one nonpolar amino acid in combination with amino- or carboxyl-terminal charged or polar amino acids. This analysis indicates that peptides with nonpolar amino acids are the most effective peptide substrates for *S. meliloti*.

### The ligand binding domain of McpU (McpU^LBD^) interacts with non-aspartate-containing dipeptides

**Fig 2:**
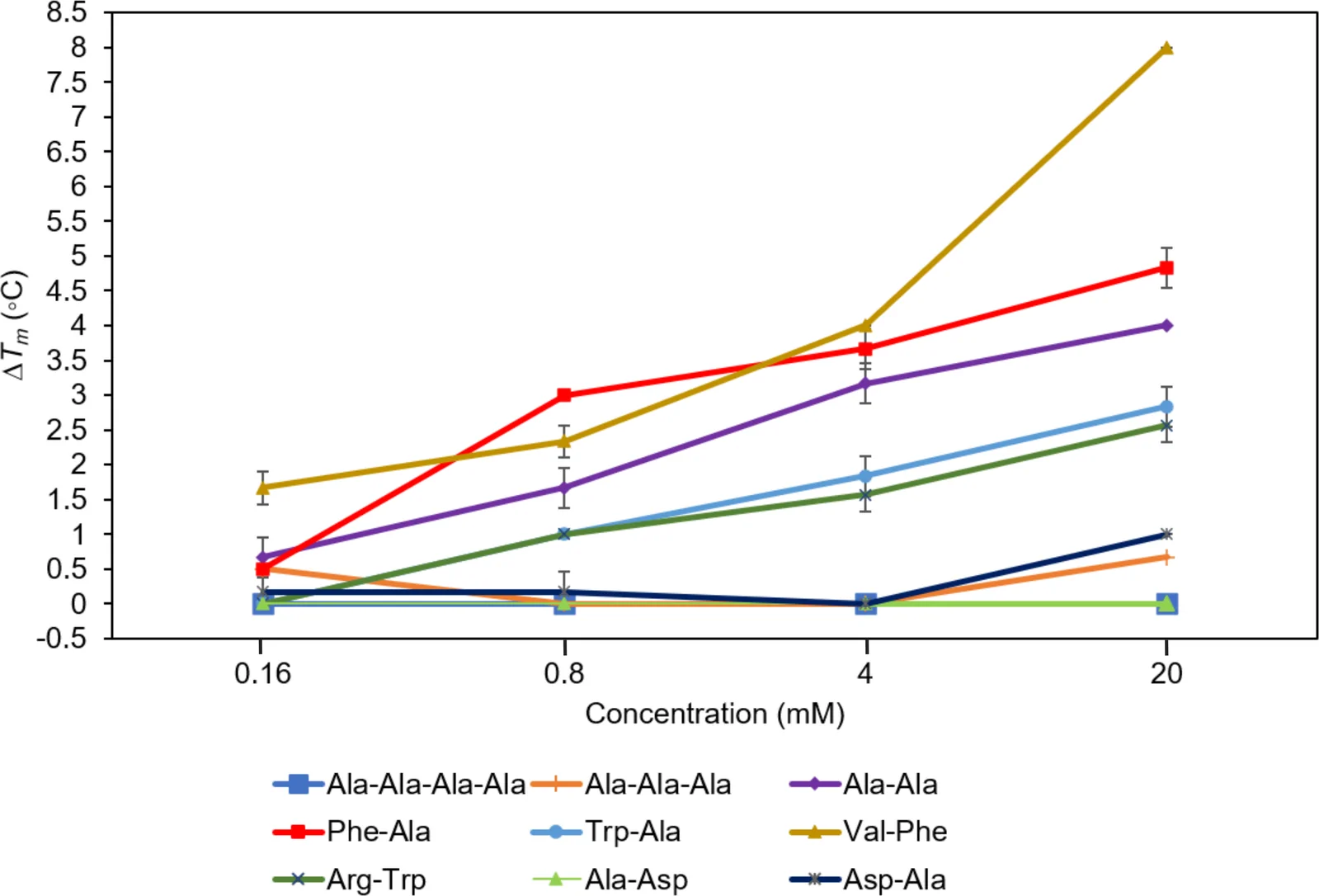
Concentration-dependent DSF assay of McpU^LBD^ with several peptides. McpU^LBD^ was tested at a concentration of 30 μM. Error bars represent the standard deviation of the mean of three independent replicates.

We reported earlier that the chemoreceptor McpU binds all proteinogenic amino acids, except Asp and Glu (22). Therefore, we hypothesized that it also binds peptides, potentially mediating a chemotaxis response to peptides in *S. meliloti*. To investigate this hypothesis, a high-throughput *in vitro* differential scanning fluorimetry (DSF) assay was employed to screen the ligand profile of isolated McpU^LBD^ using Biolog compound arrays PM6, 7, and 8. The high-throughput screen identified 103 peptides that stabilized McpU^LBD^, resulting in a thermal shift of ≥2°C (Tables S4 to S6). The threshold of ≥2°C was used as the basis for the determination of putative ligands of McpU^LBD^ (37). To verify potential peptide ligands identified from the high-throughput screen, selected peptides were custom-synthesized and subjected to DSF assays at varying concentrations. A ligand will cause increased protein thermal stability in a concentration-dependent manner. Indeed, increasing concentrations of dipeptides Ala-Ala, Arg-Trp, Phe-Ala, Trp-Ala, and Val-Phe resulted in a gradual rise of the ∆T_m_ ≥2.5°C for McpU^LBD^. However, neither dipeptides containing Asp in position 1 or 2 nor the tripeptide Ala-Ala-Ala or the tetrapeptide Ala-Ala-Ala-Ala increased the thermal stability of McpU^LBD^ (Fig. 2). This qualitative binding assay suggests that non-aspartate-containing dipeptides putatively bind to McpU^LBD^, while aspartate-containing dipeptides are not able to interact with the protein. Furthermore, tripeptides and tetrapeptides are also unable to bind to McpU^LBD^ (Fig. 2).

### The affinities of McpU^LBD^ to dipeptides and amino acids are in the same range, and the same binding sites are required for interaction

**Fig 3:**
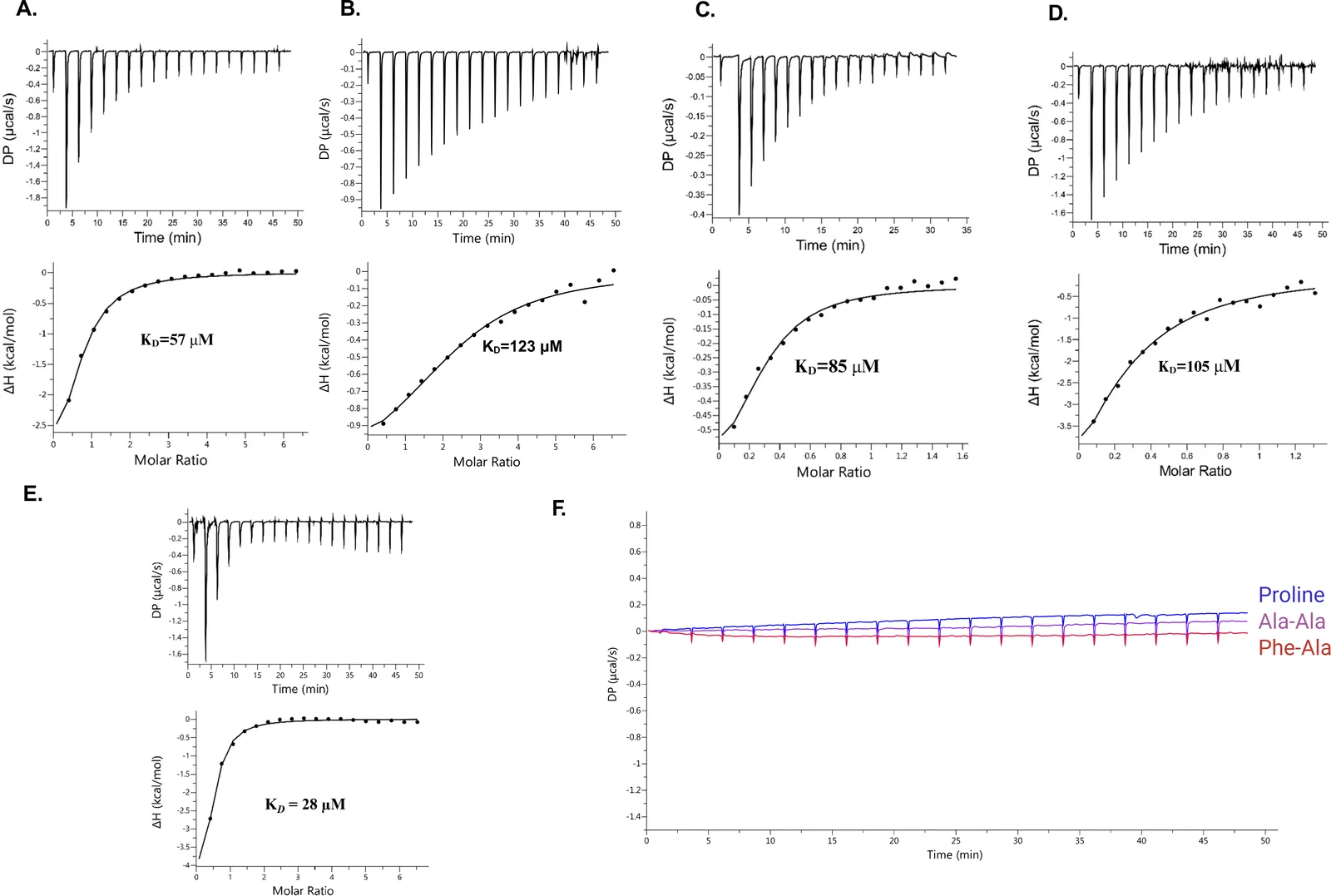
Isothermal titration calorimetry of 150 μM McpU^LBD^ or McpU^LBD (D182E)^. (**A–F**) Titration of McpU^LBD^ with 2 μL aliquots of 5 mM dipeptides. (**A**) Ala-Ala, (**B**) Arg-Trp, (**C**) Phe-Ala, (**D**) Trp-Ala, and (**E**) Val-Phe. Upper panels represent ITC raw titration data of McpU^LBD^ with respective dipeptides. The lower panels represent normalized and dilution-corrected peak areas of the raw titration data. Data were fitted using the “one set of sites fitting model” of the Malvern MicroCal PEAQ-ITC Analysis Software. (**F**) Titration of 150 µM McpU^LBD (D182E)^ with 2 μL aliquots of 0.8 mM proline and 5 mM dipeptides Ala-Ala and Phe-Ala.

Next, isothermal titration calorimetry (ITC) was used to evaluate the affinity of dipeptides to McpU^LBD^. Equilibrium dissociation constants (*K_D_*) from exothermic binding reactions for Ala-Ala, Arg-Trp, Phe-Ala, Trp-Ala, and Val-Phe were determined to be 57 , 123, 85, 105, and 28 μM, respectively (Fig. 3A through E). These values are in the same range as found for the binding of amino acids to McpU^LBD^ (21). To determine whether amino acids and dipeptides fit into the same ligand binding pocket, we performed ITC experiments with a single amino acid substitution variant of McpU^LBD^ that is unable to bind ligand. Asp-182 is an essential residue in the dCache domain located at the N-proximal end within the ligand binding pocket, and its substitution by Glu prevents amino acid binding. Titrations of McpU^LBD (D182E)^ with proline, Ala-Ala, and Phe-Ala caused no heat release and, therefore, showed no binding (Fig. 3F). In conclusion, the ITC assays reveal that both ligand types bind with a similar affinity to McpU^LBD^ and share the same binding pocket.

### The *S. meliloti* chemotaxis response to dipeptides is shifted to higher concentrations as compared to amino acids

**Fig 4:**
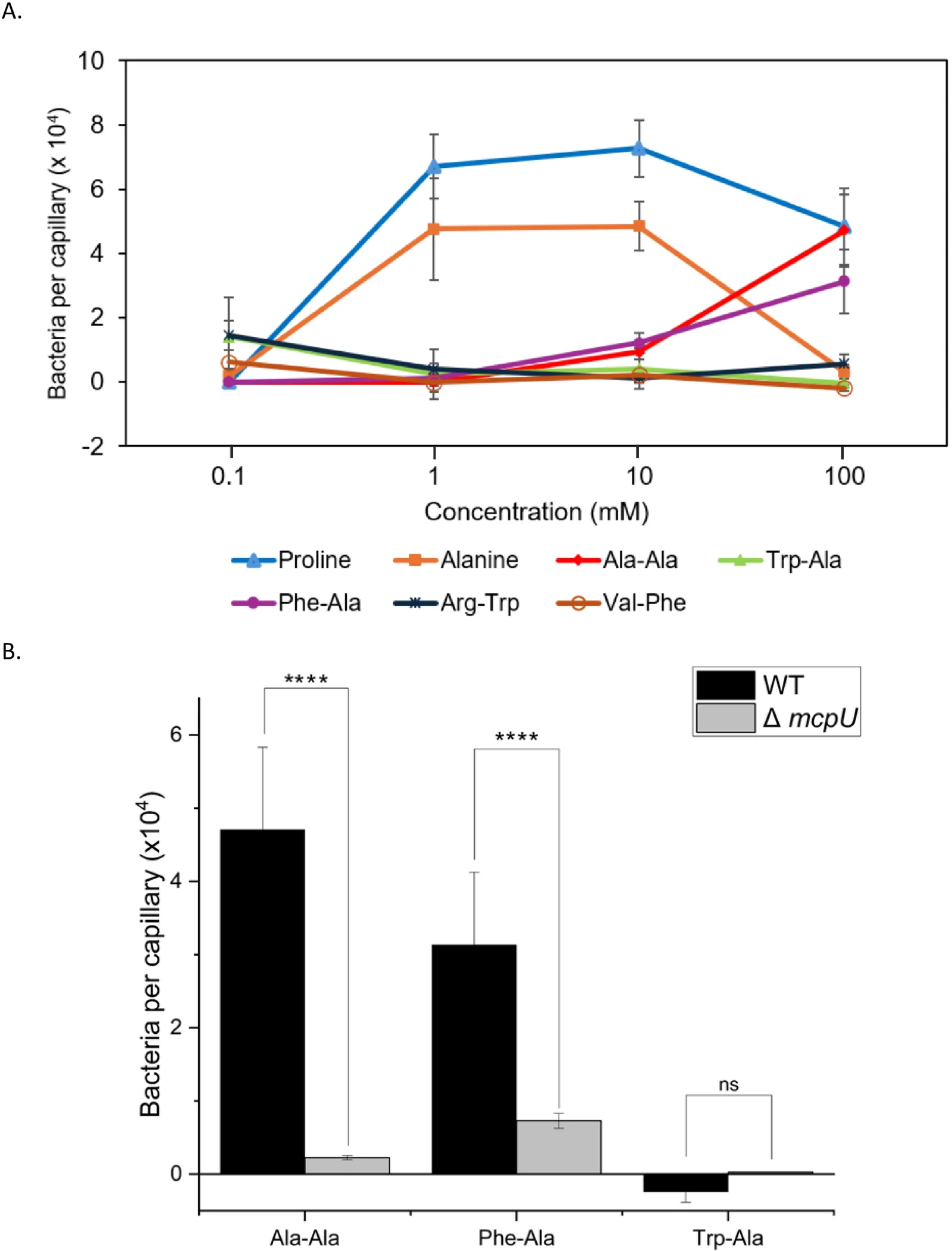
Capillary chemotaxis assay of *S. meliloti* to amino acids and dipeptides. (**A**) Chemotactic response curves of wild type to amino acids and dipeptides. (**B**) Chemotactic response of wild type and ∆*mcpU* to 100 mM dipeptides in RB. Chemotaxis data of the wild-type response are taken from **A**. The number of bacteria accumulated in three control capillaries filled with RB was subtracted from those in the test capillaries to account for the random movement of bacteria into capillaries. Asterisks denote *P* values determined by two-way ANOVA: ns, not significant; ****, *P* < 0.0001.

Since we established that dipeptides are McpU ligands, we hypothesized that *S. meliloti* would be attracted to dipeptides. Therefore, we analyzed the chemotactic behavior of *S. meliloti* wild type (RU11/001) in response to three peptides using a capillary assay (Fig. 4A). *S. meliloti* displayed positive chemotaxis to Ala-Ala and Phe-Ala. However, the response was only significant and comparable to the responses to amino acids at a peptide concentration of 100 mM. While clear responses to alanine and proline were observed at concentrations of 1 and 10 mM, dipeptides did not elicit a response at these concentrations. In addition, Trp-Ala did not elicit a chemotaxis response, even at a concentration of 100 mM (Fig. 4A). To examine whether the dipeptide response is mediated through McpU, we measured the response of the *mcpU* deletion strain (RU11/828) and found that it is not attracted to Ala-Ala, Phe-Ala, and Trp-Ala (Fig. 4B). In conclusion, *S. meliloti* exhibits a chemotaxis response to dipeptides at 10- to 100-fold higher concentrations as compared to amino acids, and this response is mediated by McpU.

### Dipeptides bind with significantly higher affinity to the solute-binding protein DppA1 as compared to McpU

**Fig 5:**
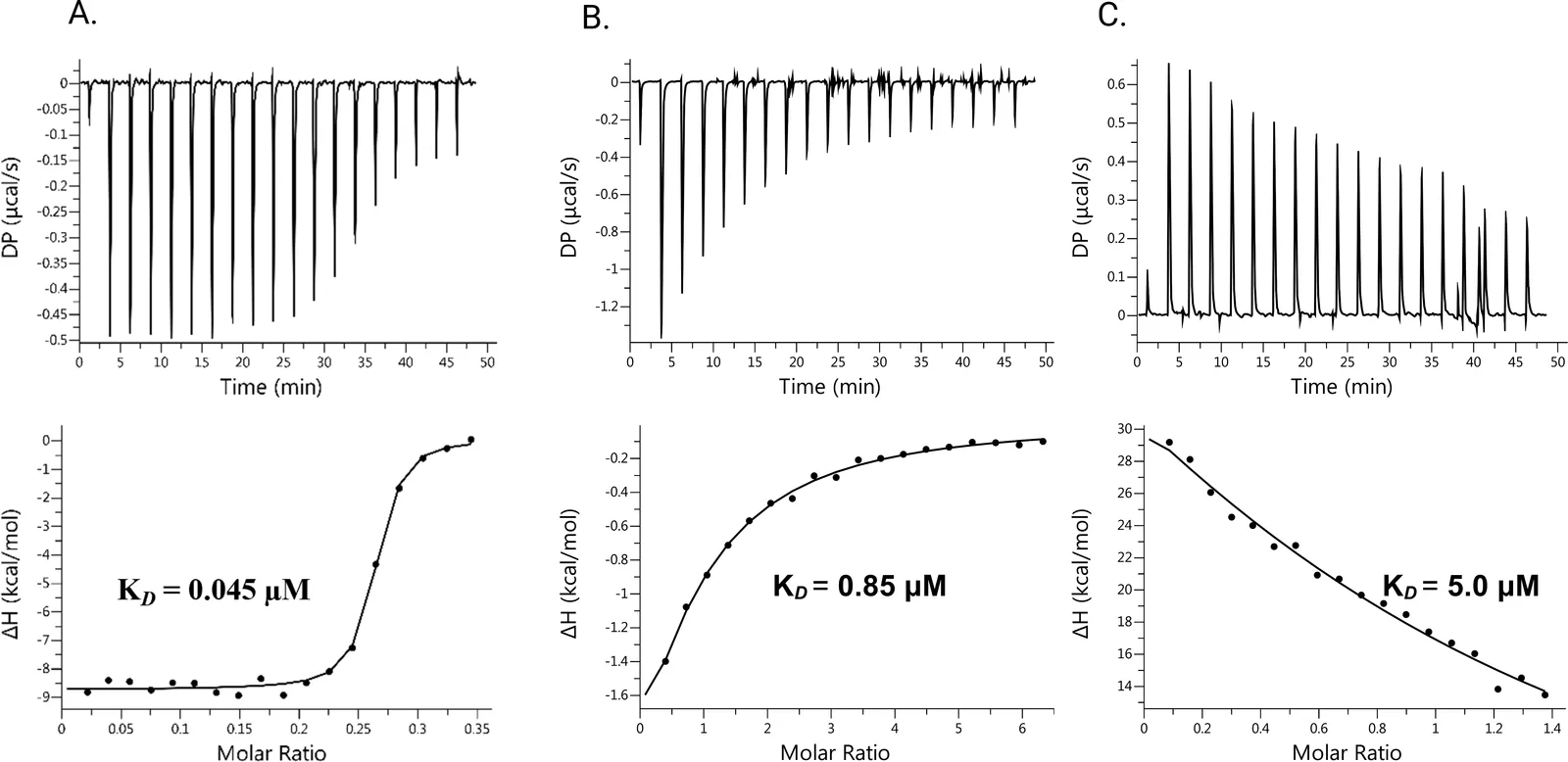
Isothermal titration calorimetry of 220 µM DppA1 with dipeptides. (**A**) 0.2 mM Ala-Ala, (**B**) 0.8 mM Phe-Ala, and (**C**) 0.8 mM Trp-Ala. Upper panels represent ITC raw titration data of DppA1 with respective dipeptides. Lower panels represent normalized and dilution-corrected peak areas of the raw titration data. Data were fitted using the “one binding site model” of the Malvern MicroCal PEAQ-ITC Analysis Software.

We hypothesized that the strength differences in amino acid and dipeptide chemotaxis are due to the different transport systems for these compounds. While amino acids are transported directly across the cytoplasmic membrane by sym- or antiporters (38), the translocation of dipeptides involves ATP-binding cassette (ABC) transporters (39). The majority of ABC transporters use solute-binding proteins (SBPs) that serve as periplasmic receptors. SBPs, including those binding dipeptides, typically have a very high affinity for their substrates. For instance, *E. coli* DppA binds the dipeptides Ala-Ala and Phe-Ala with a *K_D_* of 40 nM and 211 nM, respectively (40). If the binding affinity of *S. meliloti* SBPs for dipeptides is similarly high, we predict that they would compete with McpU for dipeptide binding in the periplasm. Therefore, we measured the binding of purified *S. meliloti* DppA1 to dipeptides via ITC and determined dissociation constants *K_D_* of 0.045, 0.85, and 5.0 μM for Ala-Ala, Phe-Ala, and Trp-Ala, respectively (Fig. 5). These values were between 1,000- and 20-fold lower than those determined for McpU^LBD^. The high affinity of DppA1 to dipeptides compared to that of McpU may explain the weak dipeptide chemotaxis response of *S. meliloti* (Fig. 4A).

### Chemotaxis of *S. meliloti* to dipeptides remains weak in the absence of two dipeptide SBPs, DppA1 and DppA2

To test whether *S. meliloti* chemotaxis to dipeptides can be enhanced by removing dipeptide-specific SBPs, we deleted *dppA1* as well as a second putative peptide-SPB encoding gene, named *dppA2* (32), and measured chemotaxis of the single and double deletion strains to Ala-Ala. For these capillary assays, we also grew cells in minimal medium with arabinose as sole carbon source to prevent any possible induction of *dpp* gene transcription by peptides present in tryptone or yeast extract containing Bromfield medium. However, we observed no significant differences in chemotactic responses to Ala-Ala for strains deleted for *dppA1* or *dppA1* and *dppA2* (Fig. S1). In conclusion, chemotaxis is only observed to certain dipeptides at a concentration of 100 mM despite the removal of two of their binding competitors in the periplasm.

### Dipeptide utilization is reduced but not abolished in the absence of *dppA1* and *dppA2*

Since chemotaxis to dipeptides was not altered in strains lacking *dppA1* and *dppA2*, we hypothesized the presence of additional dipeptide SBPs in *S. meliloti*, which would also allow bacterial growth on peptide as the sole nitrogen source. Indeed, a bioinformatics study (41) predicted the presence of several SBP-encoding genes for peptides in the *S. meliloti* genome. Thus, we analyzed whether the *dppA1dppA2* double deletion strain is capable of metabolizing (and, therefore, transporting) peptides using the Biolog growth assay. The ∆*dppA1dppA2* strain was able to utilize a total of 179 peptides out of the 282 peptides screened in comparison to 240 peptides utilized by *S. meliloti* wild type (Fig. S3A). Specifically, Ala-Ala, Phe-Ala, and Trp-Ala were metabolized by the ∆*dppA1dppA2* strain (Fig. S3; Tables S7 to S9). Taken together, DppA1 and DppA2 are essential for the uptake of only 25% of the peptides tested, which emphasizes the presence of multiple peptide transporters in *S. meliloti*. To ascertain whether the absence of these two solute-binding proteins could restore chemotaxis responses to dipeptides, we tested His-Pro and Val-Phe in the capillary chemotaxis assay. These two dipeptides were chosen because they are not utilized by the ∆*dppA1dppA2* strain. However, we did not observe a change in chemotactic behavior for this mutant. This observation suggests that the weak chemotaxis response to dipeptides extends beyond the apparent competition between solute-binding proteins and McpU in the periplasm.

### Dipeptides Ala-Ala and Trp-Ala modulate the swimming velocity of *S. meliloti*

**Fig 6:**
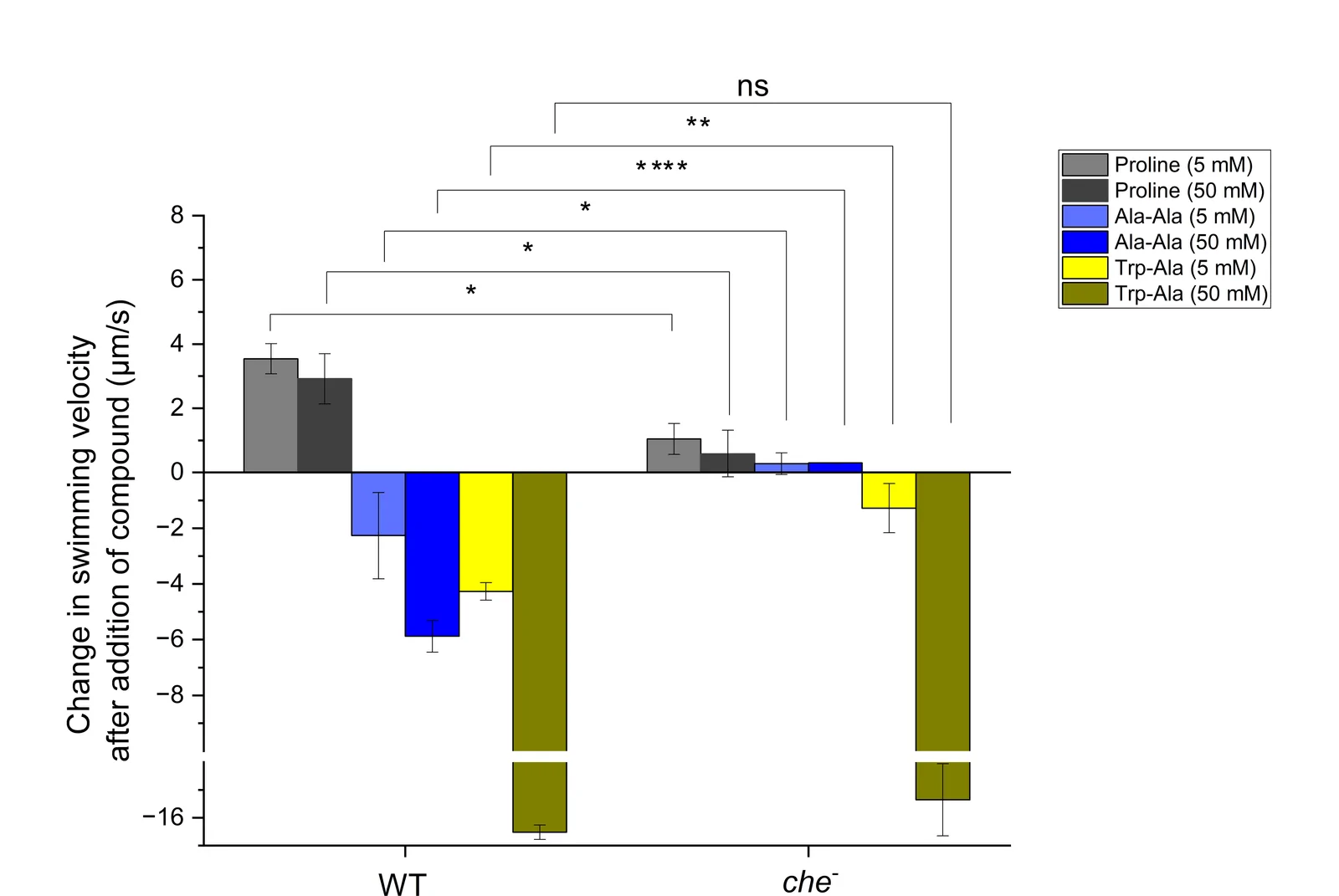
Change in swimming velocity of wild type and a strain negative for chemotaxis (*che*^−^) in the presence of 5 and 50 mM of proline, Ala-Ala, and Trp-Ala. The baseline represents the swimming velocity of each strain in RB. Values are the means and standard deviations from at least three biological replicates. Error bars represent the standard deviation of the mean of three biological replicates. Asterisks denote *P* values determined by two-way ANOVA: ns, not significant; *, *P* < 0.05; **, *P* < 0.01; and ****, *P* < 0.0001.

Due to the large number and putative nature of dipeptide SBPs, we were unable to remove all competing dipeptide receptors from the periplasm to allow for an undisturbed view of *S. meliloti* dipeptide chemotaxis. However, in addition to the generally weak chemotaxis response of *S. meliloti* to dipeptides, we noticed that even fewer wild-type cells accumulated in a capillary filled with 100 mM Trp-Ala compared to the buffer control (Fig. 4A and B). A negative chemotaxis response would explain the avoidance of *S. meliloti* to swim into a capillary filled with a repellent. We have not observed such behavior in the past and decided to further investigate this phenomenon. An independent measure for chemotaxis in *S. meliloti* is the modulation of swimming velocity in reaction to chemicals. It is known that attractant exposure causes an increase in swimming speed, a behavior also called “chemokinesis” (42). In contrast, exposure to a repellent should cause a reduction in swimming speed. To differentiate between a negative chemotaxis response and a negative effect on the physiology of *S. meliloti*, we used computerized motion analysis to measure the swimming velocities of the wild type (RU11/001) and a *che*^−^ strain (RU13/149) that lacks all chemoreceptors and plotted the changes in swimming velocity relative to that of each strain exposed to a buffer control. As expected, exposure to proline resulted in an increase in swimming velocity in wild type, which was significantly reduced in the *che*^−^ strain (43) (Fig. 6, gray bars). In contrast, responses to Ala-Ala caused a decrease in swimming velocity in wild type, which was more pronounced at higher Ala-Ala concentrations, while the swimming velocity of the *che^−^* strain was unaffected (Fig. 6, blue bars). Thus, Ala-Ala has a negative effect on the swimming speed of *S. meliloti*, and this reaction is dependent on a functional chemotaxis system. Exposure to 5 mM Trp-Ala decreased the swimming velocity of wild-type and *che*^−^ cells, but the reduction was significantly larger for the wild type, indicating a negative chemotaxis response of the wild type. In contrast, the swimming velocity for both strains decreased dramatically in the presence of 50 mM Trp-Ala, with no significant difference between both strains (Fig. 6, yellow bars). This result points to a general negative effect on the swimming speed of *S. meliloti* at higher concentrations of Trp-Ala. Taken together, these data suggest that dipeptides elicit differential chemotaxis responses in *S. meliloti* depending on their nature and concentration and that certain dipeptides can have a general, harmful effect on *S. meliloti*’s flagellar motor function.

### Dipeptides elicit a negative chemotaxis response in *S. meliloti*

**Fig 7:**
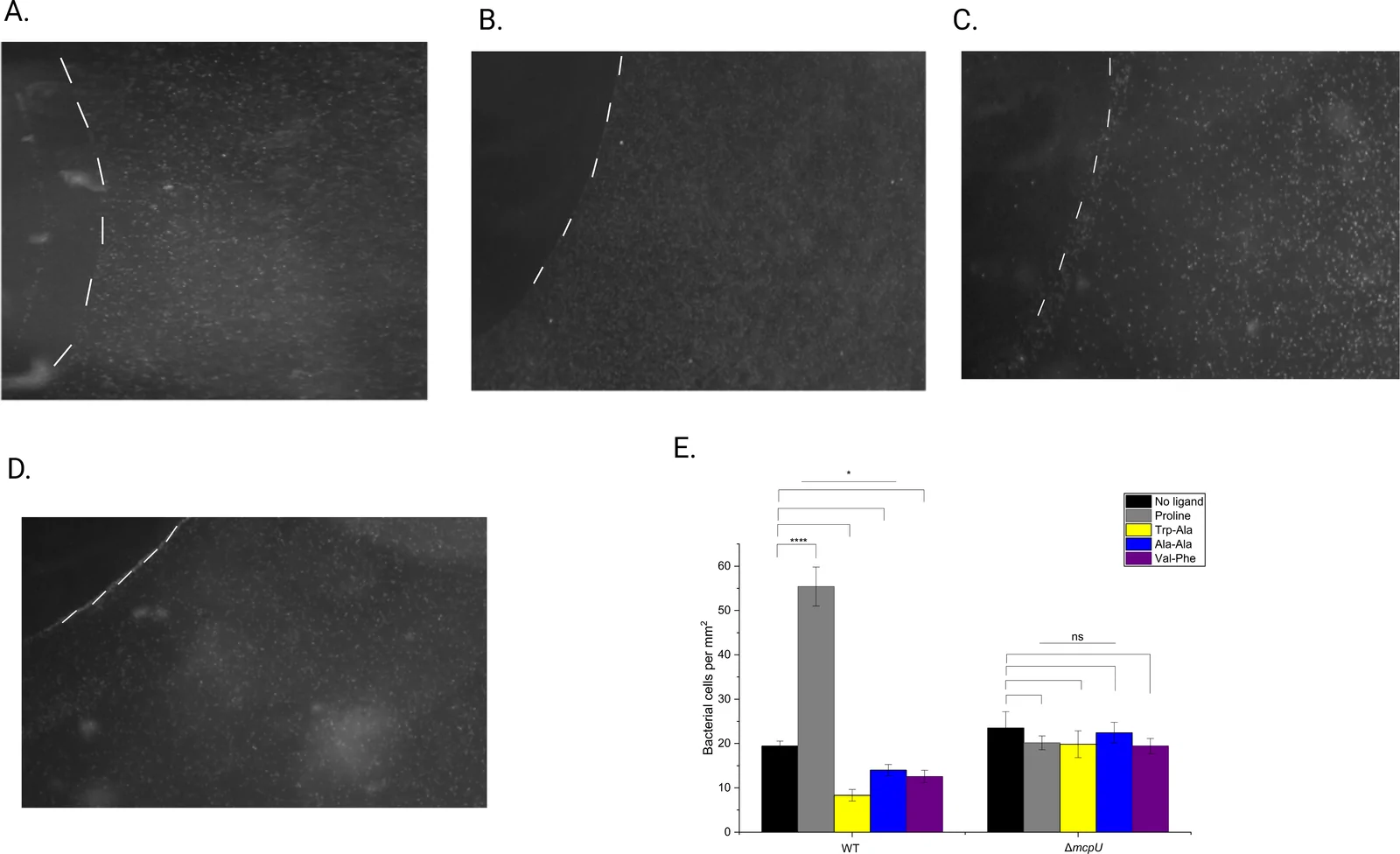
Agarose-in-plug bridge assay. Bacterial cell density per mm^2^ around an agarose plug that contained the ligand of interest was observed with a 20× objective of a phase contrast microscope 1 h after the introduction of the bacterial suspension. One plane of focus adjacent to the plug was photographed and analyzed by ImageJ software. The white dashed lines mark the edge of the agarose plug: (**A**) rhizobial basal medium as a negative control; (**B**) proline as a positive control; (**C**) Trp-Ala as a test compound; (**D**) Ala-Ala as a test compound; and (**E**) bacterial number per mm^2^ in response to ligands in agarose plugs. Error bars represent standard errors of the means of three replicates. Asterisks denote *P* values determined by two-way ANOVA: ns, not significant; *, *P* < 0.05; and ****, *P* < 0.0001.

Since this was the first observation of a chemorepellent response in *S. meliloti*, we sought to further investigate this phenomenon with the agarose-in-plug chemotaxis assay. This assay detects the movement of bacteria toward or away from chemicals within an agarose plug when viewed under a microscope (44). Bacteria accumulated around the agarose plug that contained 100 mM proline, which was clearly visible after 1 h (Fig. 7B). In contrast, bacteria moved away from plugs containing 100 mM of Ala-Ala, Trp-Ala, or Val-Phe (Fig. 7C and D). This behavior was quantified by analyzing pixel intensity of the bacterial field at a defined distance from the plug (Fig. 7E). Links to time-lapse movies of bacterial responses to the agarose-ligand plug are provided in Table S11. When analyzing the behavior of the *mcpU* deletion strain (RU11/828), bacteria did not respond to either proline or dipeptides, as they remained uniformly distributed. In conclusion, the chemoreceptor McpU mediates positive and negative chemotaxis responses to amino acids and dipeptides, respectively.

## DISCUSSION

Chemotaxis of *S. meliloti* toward amino acids, carboxylic acids, and quaternary ammonium compounds aided by specific chemoreceptors has been well established in previous studies (22, 23, 25). Among these, McpU has been characterized as a universal amino acid sensor. Alfalfa, a natural plant host of *S. meliloti*, exudes a variety of compounds in the rhizosphere to attract the bacteria toward its roots and enable the subsequent development of a symbiotic relationship to fulfill its nitrogen requirement (9, 29, 43). In an extremely competitive environment, *S. meliloti* must overcome nutritional barriers in the soil to survive. The ability to sense and move toward a variety of compounds provides an adaptive and competitive edge to surmount these limitations. The abundance of nitrogenous peptides in the soil has been well established. A significant portion of organic nitrogen in the soil exists as proteinaceous or peptidic components (34, 45). Along with chemotaxis, a broad metabolic capacity enables *S. meliloti* to adapt to various environmental and nutritional conditions (46).

Using Biolog growth assays, we revealed a broad capability of *S. meliloti* to metabolize 240 different peptides, including 76 peptides with a much higher utilization efficiency (Tables S1 to S3). Out of these efficiently utilized peptides, 75% had at least one non-polar amino acid either as amino or carboxylic constituent (Fig. 1), which showed the apparent preference toward peptides consisting of at least one nonpolar amino acid. A broad metabolic capacity toward peptide utilization is usually manifested by widespread and highly conserved ATP-binding cassette (ABC) systems. These ABC systems include importers and exporters involved in the uptake of nutrients and secretion of metabolites and are typically coupled with SBPs. *S. meliloti* has over 150 SBP and around 200 ABC encoding genes, transporting different organic and inorganic molecules including peptides, carboxylic acids, sugars, nucleic acids, and phosphates from the periplasm to cytosol (33, 41). Three ABC transporters, namely, dipeptide permease (Dpp), oligopeptide permease (Opp), and tri- and tetrapeptide permease (Ttp), have been reported to be involved in peptide transport in *S. meliloti* (35).

Following determination of broad metabolic capacity of *S. meliloti* toward peptides, we sought to investigate whether these compounds serve as chemoattractants. Since amino acid chemotaxis in *S. meliloti* is mediated by the chemoreceptor McpU (22), we explored whether McpU binds peptides. A high-throughput DSF screening assay established the interaction of dipeptides with purified McpU^LBD^ (Tables S3 to S6). Concentration-dependent DSF experiments with pure, custom-synthesized compounds confirmed binding of Ala-Ala, Arg-Trp, Phe-Ala, Trp-Ala, and Val-Phe to McpU^LBD^, while no binding was observed for dipeptides containing an aspartate as a constituting amino acid (Fig. 2). This result was not surprising, as aspartate did not bind to McpU^LBD^ or induce a chemotaxis response in *S. meliloti* wild type. McpU^LBD^ also did not bind Ala-Ala-Ala or Ala-Ala-Ala-Ala (Fig. 2). It is likely that the different structural conformations of the tri- and tetrapeptides prevent them from fitting into the McpU binding pocket. ITC experiments determined dissociation constants of Ala-Ala, Phe-Ala, and Trp-Ala in the micromolar range (Fig. 3A through E), comparable to those observed for amino acids in our earlier studies (22). Saturation of McpU^LBD^ with an amino acid prevented dipeptide binding (Fig. S1). Furthermore, the mutation of an essential residue in the proximal Cache domain of McpU (McpU^LBD(D182E)^) eliminated dipeptide binding. In conclusion, amino acids and dipeptides share the same binding pocket in McpU (Fig. 3F) (21). Surprisingly, chemotaxis capillary assays of *S. meliloti* revealed only a weak response to dipeptides in comparison to amino acids (Fig. 4A).

The unexpected weak chemotaxis response to dipeptides led to the hypothesis that other dipeptide binding proteins may interfere with McpU binding. The existence of three peptide transporters has been reported in *S. meliloti* (35). Solute-binding proteins (SBPs) have high affinities for their respective solutes (47) and can either transfer them to ABC transporters or to chemoreceptors (48, 49), thereby mediating either peptide metabolism or indirect chemotaxis. Indirect chemotaxis to dipeptides has been described in *E. coli* involving the periplasmic dipeptide binding protein DppA and chemoreceptor Tap. DppA binds dipeptides at a very high affinity with a *K*_*D*_ of 40 nM (40), and then delivers them to Tap (13). *S. meliloti* was predicted to possess 30 SBPs of the SBP_bac_5 family; the family of peptide binding SBPs (41). This high number of peptide-binding SBPs in conjunction with their typical high binding affinities may indicate that peptide binding to these proteins may significantly reduce the periplasmic concentration of free peptides. While our biochemical assays support a direct sensing mechanism of dipeptides in *S. meliloti* (Fig. 2 and 3), the presence of multiple dipeptide binding proteins with high affinities to their substrates may explain the reduced dipeptide chemotaxis in *S. meliloti*. ITC results indeed revealed a much higher affinity of DppA1 to dipeptides than McpU (20- to 1,000-fold; Fig. 5), which suggests that DppA1 competes with McpU for dipeptide binding in the periplasm. While the elimination of two of the reported dipeptide binding proteins (DppA1 and DppA2) did not alter the weak chemotaxis behavior of *S. meliloti* (Fig. S2), a literature search revealed a total of 26 putative oligopeptide-binding proteins encoded in the *S. meliloti* genome (41). The presence of additional SPBs for peptides was further validated by Biolog growth assays of the ∆*dppA1*∆*dppA2* strain, which was still able to metabolize 63% of the tested peptides (Fig. S3A; Tables S7 to S9). However, when dipeptides that were not metabolized by the ∆*dppA1*∆*dppA2* strain were analyzed in the capillary chemotaxis assay, the mutant strain did not exhibit increased chemotaxis to these peptides (Fig. S4). This result suggests that although interference of periplasmic peptide SPBs is plausible and could limit the binding of dipeptides to McpU, it seems as if the weak chemotaxis response has a different cause. The physiological significance or evolutionary reason for this phenomenon is currently unclear.

Finally, we deciphered the differential chemotaxis response of *S. meliloti* to dipeptides by measuring the swimming velocity as an indicator of chemokinesis defined as the modulation of swimming speed by a chemotaxis stimulus (42). In analogy to *E. coli*, in which binding of the phosphorylated response regulator causes a switch of motor rotation from CCW to CW, binding of the phosphorylated response regulator to the *S. meliloti* motor slows down its CW rotation. Both interactions cause the flagellar bundle to come apart and result in a tumble reaction. Consequently, *S. meliloti* responds to chemoattractants with an increase in swimming velocity (42, 43). Chemorepellents for *S. meliloti* have not been described, but are predicted to cause a decrease in swimming velocity as a response. Since chemorepellents are typically harmful to the cell, a careful comparison between the wild type and a chemotaxis-deficient (*che*^-^) strain was required for this analysis. Indeed, we measured a reduction in swimming velocity for the wild type but not the *che*^-^ strain in response to Ala-Ala (Fig. 6). This result is in agreement with the lack of bacterial accumulation in capillaries filled with 0.1, 1, or 10 mM Ala-Ala (Fig. 4A). Only capillaries filled with 100 mM Ala-Ala allowed a weak accumulation of wild-type cells. Reaction to Trp-Ala was vastly different, as it impeded swimming speed at high concentrations independent of the presence of a functional chemotaxis system, while the reduction of swimming velocity at lower concentrations was chemotaxis-dependent (Fig. 6). To characterize the differential chemotaxis responses of *S. melilot*i to dipeptides, we tested these responses using the agarose-in-plug chemotaxis assay. This assay demonstrated the chemorepellent effect of dipeptides on *S. meliloti* and confirmed that McpU mediates both positive and negative chemotaxes (Fig. 7). It remains to be elucidated how Trp-Ala exerts a negative effect, directly or indirectly, on flagellar motor function in *S. meliloti*. It should be noted that the nature of the two different assays does not allow a direct comparison of the reaction to specific compound concentrations, as cells exposed to capillaries filled with a compound react to the chemical gradient diffusing out of the capillary, whereas cells during motion analysis are directly exposed to the compound at a particular concentration.

The behavior of negative chemotaxis or chemorepulsion in bacteria is understudied (49). This is in part due to the detrimental effect of repellents on bacterial physiology, including motility, as well-established *in vivo* assays commonly used for the quantification of chemoattractant responses may not be suitable for the study of chemorepulsion. However, the direct measurement of motility and the comparison of wild-type behavior with that of a chemotaxis-deficient strain allowed the categorization of dipeptides in eliciting a differential chemotaxis response in *S. meliloti*.

In conclusion, our studies shed light on the poorly understood dynamics of dipeptide chemotaxis and its physiological relevance to *S. meliloti*’s survival in the soil. Our work revealed that the amino acid sensor McpU also binds dipeptides, with both compounds sharing a similar binding pocket and affinity. We described that *S. meliloti* can catabolize a vast array of dipeptides. The increased number of SBPs for peptides and their high affinity to their substrates present a feasible explanation for the weak chemotaxis response of *S. meliloti* to dipeptides. We also discovered that the dipeptide Trp-Ala impairs flagellar motor function. Finally, the combinatory results of chemotaxis capillary assays, swimming velocity measurements, and agarose-in-plug analyses allow the conclusion that the reaction of *S. meliloti* to dipeptides is based on their composition and concentration, resulting in differential chemotactic responses.

## MATERIALS AND METHODS

### Strains and plasmids

*S. meliloti* strains are highly motile derivatives of MVII-1 and listed in Table S10. Derivatives of *E. coli* K-12 and plasmids used for molecular techniques are also listed in Table S10.

### Media and growth conditions

*E. coli* was grown using lysogeny broth (LB) at 37°C (50). TYC medium containing 0.5% tryptone, 0.3% yeast extract (BD, Sparks, MD), and 0.13% CaCl_2_∙6H_2_O (Fisher, Fairlawn, NJ) was used to grow *S. meliloti* at 30°C. Minimal medium used for *S. meliloti* was *Rhizobium* basal medium (RB) and contained 0.1 mM NaCl, 0.01 Na_2_MoO_4_, 6.1 mM K_2_HPO_4_, 3.9 mM KH_2_PO_4_, 1 mM (NH_4_)_2_SO_4_, 1 μM FeSO_4_, 1 mM MgSO_4_, 0.1 mM CaCl_2_, 20 μg L^−1^ D-biotin, and 10 μg L^−1^ thiamin (51). Low-nutrient Bromfield plates contained (0.04% (wt/vol) tryptone, 0.01% (wt/vol) yeast extract, and 0.01% (wt/vol) CaCl_2_·2H_2_O. Ampicillin and kanamycin were used at 100 and 25 μg/mL, respectively, for *E. coli*, and streptomycin at 600 μg/mL for *S. meliloti*.

### Expression and purification of recombinant McpU^LBD^, McpU^LBD(D182E)^, and DppA1

*E. coli* M15/pREP4 was transformed with pBS373, pBS390, and pBS767 for the purification of McpU^LBD^, McpU^LBD(D182E)^, and DppA1, respectively, and expression cultures were grown in LB containing 100 µg/mL ampicillin and 25 µg/mL kanamycin at 37°C to an OD_600_ between 0.6 and 0.8 before induction with 0.3 mM isopropyl-thiogalactopyranoside (IPTG). Cultures were further incubated for 4 h at 25°C or 16 h at 16°C. Cells were harvested by centrifugation at 16,000 × *g* for 10 mins at 4 °C. Cell pellets were suspended in a binding buffer consisting of 0.5 M NaCl, 20 mM imidazole, 1 mM phenylmethane sulfonyl fluoride, and 20 mM Na P_i_, pH 7.4. The cells were lysed by two to three passages through Emulsiflex-C3 (AVESTIN, Inc., Canada). Lysates were centrifuged at 56,000 × *g* for 90 min at 4°C, followed by filtration using 0.22 μm filters. The lysate was then applied to a 5 mL Ni-NTA affinity column (GE Healthcare) equilibrated with binding buffer at a flow rate of 5 mL/min and washed with five column volumes. Elution buffer containing 0.5 M imidazole, 0.5 M NaCl, and 20 mM NaP_i_, pH 7.4, was applied to the column in an increasing linear gradient. Protein elution was monitored by UV absorbance at a wavelength of 280 nm and confirmed by SDS-PAGE. Pooled fractions containing protein were further purified by size exclusion chromatography using a HiPrep 26/60 Sephacryl S-200 HR column (GE Healthcare) in 100 mM NaCl and 50 mM HEPES, pH 7.4, at a flow rate of 1 mL/min for DSF and ITC experiments. When appropriate, the protein was concentrated using an Amicon Ultrafiltration System and 10-kDa regenerated cellulose ultrafiltration discs (Millipore, Billerica, MA).

### Biolog growth assays with peptides

Dipeptide sources containing Biolog phenotypic microarray plates, PM6, PM7, and PM8 (Biolog, Hayward, CA), were employed to investigate *S. meliloti* metabolism of peptides as nitrogen sources. *S. meliloti* WT and ∆*dppA1dppA2* were grown on BUG + B agar plates at 30°C for 48 h. Cells were transferred to a capped tube containing 16 mL of 1× IF-0 inoculating fluid, and the OD_600_ was adjusted to a transmittance of 42%. Next, 15 mL of the prepared cell suspension was added to 75 mL of 1× IF-0 and dye mix A to obtain a final transmittance of 85%, followed by the addition of 2 M sodium succinate. One hundred microliters of the cell suspension was inoculated into each well of the PM6, PM7, and PM8 microplates, followed by incubation at 30°C for 72 h. Growth was indicated by a reduction of tetrazolium dye leading to the formation of formazan as indicated by an intensive purple color (Fig. S1). Microplates were measured at an absorbance of 590 nm using a microarray plate reader to determine the color change as a direct correlation of bacterial growth.

### Qualitative binding assays of McpU^LBD^ to peptides

To investigate the potential interactions between McpU and peptides, an *in vitro* protein-ligand interaction experiment was performed using differential scanning fluorimetry (DSF). For this, peptides containing Biolog plates PM6, PM7, and PM8 were used. Compounds in PM6, PM7, and PM8 microplates (Biolog, Hayward, CA) were dissolved in 50 µL DSF buffer (100 mM NaCl, 50 mM HEPES, pH 7.4) by placing on an orbital shaker for 20 min prior to use. Aliquots of 2.5 µL of compound solutions were transferred into individual wells of a 96-well plate containing a Master Mix of 22.5 µL of 30 µM McpU^LBD^ and 5× SYPRO Orange in DSF buffer. A thermal shift (Δ*T_m_*) of ≥2°C was used as the basis for the determination of the putative binding profile of McpU^LBD^ to the various peptides. Selected peptides were custom-synthesized with >99% purity (Bachem Americas, Inc., Torrance, CA) and dissolved in DSF buffer for pure-compound DSF assays. The Δ*T_m_* was determined by subtracting the *T_m_* of the control well containing no ligand from the *T_m_* of each test well.

### Isothermal titration calorimetry (ITC)

Direct binding analysis was performed with a MicroCal PEAQ-ITC (Malvern Panalytical, Ltd.). Three hundred microliters of protein samples was loaded into the sample cell and titrated with 5 mM dipeptide solution from the syringe. All ITC experiments were performed at 25°C. Protein and ligand solutions were degassed at 27°C before loading them into the MicroCal PEAQ-ITC. To obtain baseline titrations and for reference subtraction, dipeptides were titrated into the buffer without protein. The dissociation constant (KD) was determined from heat changes during titration of the ligand with the protein using the MicroCal PEAQ-ITC analysis software “one binding sites” model.

### Capillary assays

Capillary assays were performed as originally described by Adler (52), with minor modifications for *S. meliloti* (24). Motile *S. meliloti* cells were obtained by diluting stationary phase TYC cultures into 10 mL of *Rhizobium* basal medium (RB) overlain onto Bromfield agar plates and incubated at 30°C for 15 h. Cells were harvested between an OD_600_ of 0.15 and 0.18 and sedimented by centrifugation at 3,000 × *g* for 5 min before being suspended to a final OD_600_ of 0.15 in RB medium. Next, 350 μL of bacterial suspension was placed into a pond formed from a U-shaped glass tube between two glass plates. Microcaps glass capillaries (1.0 μL, Drummond Scientific, Broomall, PA) were sealed at one end over a flame and placed into 1.5 mL microcentrifuge tubes containing ligands ranging from 0.1 to 100 mM prepared in RB medium. These were centrifuged at 2,500 × *g* for 30 s to allow the solution to fill the capillary. Capillaries were placed into the U-shaped bacterial ponds and incubated at room temperature for 2 h. The capillaries were then removed and broken at the sealed tip, and their contents were collected into 999 μL RB. Fifty microliters of the collected samples was plated in duplicates onto TYC-containing streptomycin plates using a spiral plater (Don Whitley Scientific, United Kingdom), and colonies were counted after 72 h/3 days of growth using a spiral reader. The counts of a control capillary were subtracted from all test capillaries to account for accumulation due to random movement of bacteria into the capillary. Three technical replicates were performed for each of three biological replicates, and values are represented as the mean number of bacterial cells per capillary.

### Swimming velocity analysis

*S. meliloti* strains were grown on RB-overlaid Bromfield plates to an OD_600_ of 0.15-0.18 at 30°C. Cells were initially diluted to an OD_600_ of 0.1 in RB medium and used for the experiment. Ten microliters of bacterial cells at OD_600_ 0.1 was mixed with an equal volume (10 µL) of either RB (control) or 10 and 100 mM of proline, Ala-Ala, and Trp-Ala to obtain a final OD_600_ of 0.05. Five-second videos were captured with a phase contrast microscope with a 40× objective (Nikon Eclipse E600) and a custom Nikon CMOS camera (The Imaging Source, Charlotte, NC, USA) and edited using ImageJ (53). Swimming velocities were analyzed using the TumbleScore program in MATLAB 2025a (54). Statistical significance was determined by a two-way analysis of variance (ANOVA).

### Agarose-in-plug bridge assay

Agarose-in-plug-bridge assays were performed as described by Korolik and Ottemann (44), with minor modifications. *S. meliloti* cells were grown overnight at 30°C on RB-overlaid Bromfield plates. Cells were washed and suspended in RB medium to a final OD_600_ of 0.7–1.0. Agarose plugs were prepared by heating Nusieve agarose at a final concentration of 2% with ligands at 70°C until the agarose melted and cooled at 65°C for immediate use. Twelve microliters of melted agarose-ligand solution was pipetted onto a glass slide with two cover slips 1.5 cm apart on both sides of the agarose plug, and a cover glass was placed on top of the agarose. Hundred microliters of washed *S. meliloti* cells was pipetted under the top glass cover slip and observed with a 20× objective for areas adjacent to the agarose plug for up to 1 hour after initiating the assay.

## SUPPLEMENTAL MATERIAL

Supplemental material mbio.01739-26-s0001.docx Supplemental figures and tables.

ASM does not own the copyrights to Supplemental Material that may be linked to, or accessed through, an article. The authors have granted ASM a non-exclusive, world-wide license to publish the Supplemental Material files. Please contact the corresponding author directly for reuse.
