# Supplementary material for: The Sinorhizobium meliloti chemoreceptor McpU mediates positive and negative chemotaxis responses to dipeptides and competes with periplasmic solute-binding proteins for ligand binding
Source: mBio. 2026 Aug 20;17(9):e01739-26. doi: 10.1128/mbio.01739-26 (PMC13556227; doi:10.1128/mbio.01739-26)
Supplement: Supplementary file 1 [file mbio.01739-26-s0001.docx]

SUPPLEMENTAL MATERIAL TO

**The *Sinorhizobium meliloti* chemoreceptor McpU mediates positive and negative chemotaxis responses to dipeptides and competes with periplasmic solute-binding proteins for ligand binding**

Foster K. Agyei, Nisha Dhiman, and Birgit E. Scharf

Table S1. Biolog growth assay of *S. meliloti* WT with phenotypic microarray plates 6 (PM6 peptide nitrogen sources). Peptides in wells with an average absorbance (590 nm) ≥ 0.2 were considered positive for utilization by *S. meliloti*.

| Well | Sample | Absorbance (590) 1 | Absorbance (590) 2 | Average Absorbance |
| --- | --- | --- | --- | --- |
| A01 | Negative control | 0.144 | 0.16 | **0.15** |
| A02 | Positive control | 0.42 | 0.232 | **0.33** |
| A03 | Ala-Ala | 0.377 | 0.32 | **0.35** |
| A04 | Ala-Arg | 0.385 | 0.307 | **0.35** |
| A05 | Ala-Asn | 0.488 | 0.368 | **0.43** |
| A06 | Ala-Glu | 0.398 | 0.365 | **0.38** |
| A07 | Ala-Gly | 0.503 | 0.417 | **0.46** |
| A08 | Ala-His | 0.522 | 0.447 | **0.49** |
| A09 | Ala-Leu | 0.382 | 0.315 | **0.35** |
| A10 | Ala-Lys | 0.446 | 0.355 | **0.40** |
| A11 | Ala-Phe | 0.349 | 0.314 | **0.33** |
| A12 | Ala-Pro | 0.31 | 0.305 | **0.31** |
| B01 | Ala-Ser | 0.403 | 0.336 | **0.37** |
| B02 | Ala-Thr | 0.241 | 0.174 | **0.21** |
| B03 | Ala-Trp | 0.589 | 0.52 | **0.56** |
| B04 | Ala-Tyr | 0.42 | 0.384 | **0.40** |
| B05 | Arg-Ala | 0.398 | 0.373 | **0.39** |
| B06 | Arg-Arg | 0.466 | 0.405 | **0.44** |
| B07 | Arg-Asp | 0.378 | 0.344 | **0.36** |
| B08 | Arg-Gln | 0.416 | 0.358 | **0.39** |
| B09 | Arg-Glu | 0.363 | 0.381 | **0.37** |
| B10 | Arg-Ile | 0.317 | 0.312 | **0.32** |
| B11 | Arg-Leu | 0.355 | 0.343 | **0.35** |
| B12 | Arg-Lys | 0.377 | 0.337 | **0.36** |
| C01 | Arg-Met | 0.314 | 0.319 | **0.32** |
| C02 | Arg-Phe | 0.298 | 0.254 | **0.28** |
| C03 | Arg-Ser | 0.503 | 0.465 | **0.48** |
| C04 | Arg-Trp | 0.734 | 0.632 | **0.68** |
| C05 | Arg-Tyr | 0.347 | 0.326 | **0.34** |
| C06 | Arg-Val | 0.313 | 0.308 | **0.31** |
| C07 | Asn-Glu | 0.402 | 0.373 | **0.39** |
| C08 | Asn-Val | 0.317 | 0.281 | **0.30** |
| C09 | Asp-Asp | 0.324 | 0.314 | **0.32** |
| C10 | Asp-Glu | 0.287 | 0.245 | **0.27** |
| C11 | Asp-Leu | 0.316 | 0.296 | **0.31** |
| C12 | Asp-Lys | 0.478 | 0.397 | **0.44** |
| D01 | Asp-Phe | 0.214 | 0.206 | **0.21** |
| D02 | Asp-Trp | 0.358 | 0.266 | **0.31** |
| D03 | Asp-Val | 0.303 | 0.269 | **0.29** |
| D04 | Cys-Gly | 0.755 | 0.755 | **0.76** |
| D05 | Gln-Gln | 0.445 | 0.372 | **0.41** |
| D06 | Gln-Gly | 0.428 | 0.368 | **0.40** |
| D07 | Glu-Asp | 0.293 | 0.26 | **0.28** |
| D08 | Glu-Glu | 0.287 | 0.276 | **0.28** |
| D09 | Glu-Gly | 0.304 | 0.268 | **0.29** |
| D10 | Glu-Ser | 0.452 | 0.437 | **0.45** |
| D11 | Glu-Trp | 0.43 | 0.37 | **0.40** |
| D12 | Glu-Tyr | 0.337 | 0.363 | **0.35** |
| E01 | Glu-Val | 0.271 | 0.24 | **0.26** |
| E02 | Gly-Ala | 0.408 | 0.369 | **0.39** |
| E03 | Gly-Arg | 0.293 | 0.282 | **0.29** |
| E04 | Gly-Cys | 0.52 | 0.532 | **0.53** |
| E05 | Gly-Gly | 0.257 | 0.223 | **0.24** |
| E06 | Gly-His | 0.438 | 0.406 | **0.42** |
| E07 | Gly-Leu | 0.322 | 0.301 | **0.31** |
| E08 | Gly-Lys | 0.4 | 0.372 | **0.39** |
| E09 | Gly-Met | 0.23 | 0.242 | **0.24** |
| E10 | Gly-Phe | 0.281 | 0.273 | **0.28** |
| E11 | Gly-Pro | 0.267 | 0.216 | **0.24** |
| E12 | Gly-Ser | 0.424 | 0.403 | **0.41** |
| F01 | Gly-Thr | 0.313 | 0.248 | **0.28** |
| F02 | Gly-Trp | 0.44 | 0.369 | **0.41** |
| F03 | Gly-Tyr | 0.324 | 0.29 | **0.31** |
| F04 | Gly-Val | 0.25 | 0.209 | **0.23** |
| F05 | His-Asp | 0.438 | 0.377 | **0.41** |
| F06 | His-Gly | 0.372 | 0.35 | **0.36** |
| F07 | His-Leu | 0.253 | 0.247 | **0.25** |
| F08 | His-Lys | 0.386 | 0.371 | **0.38** |
| F09 | His-Met | 0.204 | 0.188 | **0.19** |
| F10 | His-Pro | 0.478 | 0.438 | **0.46** |
| F11 | His-Ser | 0.437 | 0.377 | **0.41** |
| F12 | His-Trp | 0.493 | 0.465 | **0.48** |
| G01 | His-Tyr | 0.451 | 0.422 | **0.44** |
| G02 | His-Val | 0.285 | 0.25 | **0.27** |
| G03 | Ile-Ala | 0.244 | 0.251 | **0.25** |
| G04 | Ile-Arg | 0.329 | 0.344 | **0.34** |
| G05 | Ile-Gln | 0.255 | 0.26 | **0.26** |
| G06 | Ile-Gly | 0.249 | 0.208 | **0.23** |
| G07 | Ile-His | 0.329 | 0.299 | **0.31** |
| G08 | Ile-Ile | 0.205 | 0.198 | **0.20** |
| G09 | Ile-Met | 0.189 | 0.194 | **0.19** |
| G10 | Ile-Phe | 0.187 | 0.198 | **0.19** |
| G11 | Ile-Pro | 0.234 | 0.176 | **0.21** |
| G12 | Ile-Ser | 0.311 | 0.317 | **0.31** |
| H01 | Ile-Trp | 0.507 | 0.487 | **0.50** |
| H02 | Ile-Tyr | 0.246 | 0.371 | **0.31** |
| H03 | Ile-Val | 0.22 | 0.229 | **0.23** |
| H04 | Leu-Ala | 0.333 | 0.338 | **0.34** |
| H05 | Leu-Arg | 0.346 | 0.37 | **0.36** |
| H06 | Leu-Asp | 0.39 | 0.405 | **0.39** |
| H07 | Leu-Glu | 0.28 | 0.333 | **0.31** |
| H08 | Leu-Gly | 0.317 | 0.333 | **0.33** |
| H09 | Leu-Ile | 0.267 | 0.276 | **0.27** |
| H10 | Leu-Leu | 0.262 | 0.278 | **0.27** |
| H11 | Leu-Met | 0.2 | 0.256 | **0.23** |
| H12 | Leu-Phe | 0.296 | 0.338 | **0.32** |

Table S2. Biolog growth assay of *S. meliloti* WT with phenotypic microarray plates 7 (PM7 peptide nitrogen sources). Peptides in wells with an average absorbance (590 nm) ≥ 0.2 were considered positive for utilization by *S. meliloti*.

| Well | Sample | Absorbance (590) 1 | Absorbance (590) 2 | | Average Absorbance |
| --- | --- | --- | --- | --- | --- |
| A01 | Negative control | 0.183 | 0.144 | **0.16** | |
| A02 | Positive control | 0.382 | 0.311 | **0.35** | |
| A03 | Leu-Ser | 0.467 | 0.394 | **0.43** | |
| A04 | Leu-Trp | 0.538 | 0.461 | **0.50** | |
| A05 | Leu-Val | 0.269 | 0.234 | **0.25** | |
| A06 | Lys-Ala | 0.258 | 0.256 | **0.26** | |
| A07 | Lys-Arg | 0.339 | 0.366 | **0.35** | |
| A08 | Lys-Glu | 0.247 | 0.257 | **0.25** | |
| A09 | Lys-Ile | 0.267 | 0.261 | **0.26** | |
| A10 | Lys-Leu | 0.314 | 0.311 | **0.31** | |
| A11 | Lys-Lys | 0.354 | 0.307 | **0.33** | |
| A12 | Lys-Phe | 0.294 | 0.261 | **0.28** | |
| B1 | Lys-Pro | 0.209 | 0.177 | **0.19** | |
| B2 | Lys-Ser | 0.288 | 0.25 | **0.27** | |
| B3 | Lys-Thr | 0.213 | 0.203 | **0.21** | |
| B4 | Lys-Trp | 0.297 | 0.302 | **0.30** | |
| B5 | Lys-Tyr | 0.359 | 0.337 | **0.35** | |
| B6 | Lys-Val | 0.201 | 0.188 | **0.19** | |
| B7 | Met-Arg | 0.27 | 0.204 | **0.24** | |
| B8 | Met-Asp | 0.142 | 0.149 | **0.15** | |
| B9 | Met-Gln | 0.176 | 0.16 | **0.17** | |
| B10 | Met-Glu | 0.162 | 0.134 | **0.15** | |
| B11 | Met-Gly | 0.317 | 0.197 | **0.26** | |
| B12 | Met-His | 0.216 | 0.211 | **0.21** | |
| C1 | Met-Ile | 0.195 | 0.167 | **0.18** | |
| C2 | Met-Leu | 0.209 | 0.175 | **0.19** | |
| C3 | Met-Lys | 0.228 | 0.179 | **0.20** | |
| C4 | Met-Met | 0.139 | 0.153 | **0.15** | |
| C5 | Met-Phe | 0.132 | 0.131 | **0.13** | |
| C6 | Met-Pro | 0.137 | 0.137 | **0.14** | |
| C7 | Met-Trp | 0.287 | 0.331 | **0.31** | |
| C8 | Met-Val | 0.174 | 0.167 | **0.17** | |
| C9 | Phe-Ala | 0.244 | 0.199 | **0.22** | |
| C10 | Phe-Gly | 0.295 | 0.257 | **0.28** | |
| C11 | Phe-Ile | 0.186 | 0.164 | **0.18** | |
| C12 | Phe-Phe | 0.076 | 0.084 | **0.08** | |
| D1 | Phe-Pro | 0.162 | 0.154 | **0.16** | |
| D2 | Phe-Ser | 0.256 | 0.231 | **0.24** | |
| D3 | Phe-Trp | 0.125 | 0.1 | **0.11** | |
| D4 | Pro-Ala | 0.25 | 0.221 | **0.24** | |
| D5 | Pro-Asp | 0.218 | 0.189 | **0.20** | |
| D6 | Pro-Gln | 0.341 | 0.284 | **0.31** | |
| D7 | Pro-Gly | 0.221 | 0.192 | **0.21** | |
| D8 | Pro-Hyp | 0.214 | 0.182 | **0.19** | |
| D9 | Pro-Leu | 0.27 | 0.232 | **0.25** | |
| D10 | Pro-Phe | 0.214 | 0.224 | **0.22** | |
| D11 | Pro-Pro | 0.183 | 0.154 | **0.17** | |
| D12 | Pro-Tyr | 0.281 | 0.272 | **0.28** | |
| E1 | Ser-Ala | 0.379 | 0.398 | **0.39** | |
| E2 | Ser-Gly | 0.382 | 0.316 | **0.35** | |
| E3 | Ser-His | 0.422 | 0.388 | **0.41** | |
| E4 | Ser-Leu | 0.323 | 0.289 | **0.31** | |
| E5 | Ser-Met | 0.19 | 0.163 | **0.18** | |
| E6 | Ser-Phe | 0.276 | 0.233 | **0.26** | |
| E7 | Ser-Pro | 0.22 | 0.191 | **0.21** | |
| E8 | Ser-Ser | 0.228 | 0.179 | **0.20** | |
| E9 | Ser-Tyr | 0.295 | 0.279 | **0.29** | |
| E10 | Ser-Val | 0.237 | 0.202 | **0.22** | |
| E11 | Thr-Ala | 0.177 | 0.174 | **0.18** | |
| E12 | Thr-Arg | 0.298 | 0.254 | **0.28** | |
| F1 | Thr-Glu | 0.213 | 0.175 | **0.19** | |
| F2 | Thr-Gly | 0.205 | 0.161 | **0.18** | |
| F3 | Thr-Leu | 0.239 | 0.188 | **0.21** | |
| F4 | Thr-Met | 0.165 | 0.154 | **0.16** | |
| F5 | Thr-Pro | 0.157 | 0.143 | **0.15** | |
| F6 | Trp-Ala | 0.386 | 0.373 | **0.38** | |
| F7 | Trp-Arg | 0.461 | 0.483 | **0.47** | |
| F8 | Trp-Asp | 0.294 | 0.265 | **0.28** | |
| F9 | Trp-Glu | 0.242 | 0.239 | **0.24** | |
| F10 | Trp-Gly | 0.395 | 0.422 | **0.41** | |
| F11 | Trp-Leu | 0.302 | 0.278 | **0.29** | |
| F12 | Trp-Lys | 0.42 | 0.338 | **0.38** | |
| G1 | Trp-Phe | 0.187 | 0.318 | **0.25** | |
| G2 | Trp-Ser | 0.421 | 0.406 | **0.41** | |
| G3 | Trp-Trp | 0.289 | 0.37 | **0.33** | |
| G4 | Trp-Tyr | 0.177 | 0.169 | **0.17** | |
| G5 | Tyr-Ala | 0.279 | 0.226 | **0.25** | |
| G6 | Tyr-Gln | 0.2 | 0.183 | **0.192]** | |
| G7 | Tyr-Glu | 0.193 | 0.158 | **0.18** | |
| G8 | Tyr-Gly | 0.287 | 0.249 | **0.27** | |
| G9 | Tyr-His | 0.309 | 0.312 | **0.31** | |
| G10 | Tyr-Leu | 0.213 | 0.172 | **0.19** | |
| G11 | Tyr-Lys | 0.304 | 0.289 | **0.29** | |
| G12 | Tyr-Phe | 0.188 | 0.216 | **0.20** | |
| H1 | Tyr-Trp | 0.259 | 0.307 | **0.28** | |
| H2 | Tyr-Tyr | 0.233 | 0.184 | **0.21** | |
| H3 | Val-Arg | 0.263 | 0.216 | **0.24** | |
| H4 | Val-Asn | 0.324 | 0.241 | **0.28** | |
| H5 | Val-Asp | 0.192 | 0.199 | **0.19** | |
| H6 | Val-Gly | 0.239 | 0.219 | **0.23** | |
| H7 | Val-His | 0.25 | 0.204 | **0.23** | |
| H8 | Val-Ile | 0.182 | 0.166 | **0.17** | |
| H9 | Val-Leu | 0.205 | 0.188 | **0.19** | |
| H10 | Val-Tyr | 0.212 | 0.173 | **0.19** | |
| H11 | Val-Val | 0.168 | 0.164 | **0.17** | |
| H12 | γ-Glu-Gly | 0.25 | 0.247 | **0.25** | |

Table S3. Biolog growth assay of *S. meliloti* WT with phenotypic microarray plates 8 (PM8 peptide nitrogen sources). Peptides in wells with an average absorbance (590 nm) ≥ 0.2 were considered positive for utilization by *S. meliloti*.

| Well | Sample | Absorbance (590) 1 | Absorbance (590) 2 | Average Absorbance |
| --- | --- | --- | --- | --- |
| A01 | Negative control | 0.172 | 0.159 | **0.17** |
| A02 | Positive control | 0.585 | 0.566 | **0.58** |
| A03 | Ala-Asp | 0.489 | 0.452 | **0.47** |
| A04 | Ala-Gln | 0.677 | 0.656 | **0.67** |
| A05 | Ala-Ile | 0.46 | 0.444 | **0.45** |
| A06 | Ala-Met | 0.36 | 0.36 | **0.36** |
| A07 | Ala-Val | 0.41 | 0.403 | **0.41** |
| A08 | Asp-Ala | 0.417 | 0.421 | **0.42** |
| A09 | Asp-Gln | 0.464 | 0.454 | **0.46** |
| A10 | Asp-Gly | 0.413 | 0.437 | **0.43** |
| A11 | Glu-Ala | 0.607 | 0.613 | **0.61** |
| A12 | Gly-Asn | 0.611 | 0.589 | **0.60** |
| B1 | Gly-Asp | 0.456 | 0.438 | **0.45** |
| B2 | Gly-Ile | 0.473 | 0.437 | **0.46** |
| B3 | His-Ala | 0.541 | 0.531 | **0.54** |
| B4 | His-Glu | 0.617 | 0.594 | **0.61** |
| B5 | His-His | 0.542 | 0.505 | **0.52** |
| B6 | Ile-Asn | 0.481 | 0.493 | **0.49** |
| B7 | Ile-Leu | 0.378 | 0.387 | **0.38** |
| B8 | Leu-Asn | 0.566 | 0.544 | **0.56** |
| B9 | Leu-His | 0.527 | 0.517 | **0.52** |
| B10 | Leu-Pro | 0.409 | 0.433 | **0.42** |
| B11 | Leu-Tyr | 0.43 | 0.417 | **0.42** |
| B12 | Lys-Asp | 0.349 | 0.364 | **0.36** |
| C1 | Lys-Gly | 0.522 | 0.488 | **0.51** |
| C2 | Lys-Met | 0.315 | 0.277 | **0.29** |
| C3 | Met-Thr | 0.301 | 0.292 | **0.29** |
| C4 | Met-Tyr | 0.621 | 0.522 | **0.57** |
| C5 | Phe-Asp | 0.352 | 0.307 | **0.33** |
| C6 | Phe-Glu | 0.335 | 0.334 | **0.34** |
| C7 | Gln-Glu | 0.621 | 0.597 | **0.61** |
| C8 | Phe-Met | 0.212 | 0.226 | **0.22** |
| C9 | Phe-Tyr | 0.292 | 0.287 | **0.29** |
| C10 | Phe-Val | 0.297 | 0.27 | **0.28** |
| C11 | Pro-Arg | 0.591 | 0.62 | **0.61** |
| C12 | Pro-Asn | 0.581 | 0.567 | **0.57** |
| D1 | Pro-Glu | 0.438 | 0.436 | **0.44** |
| D2 | Pro-Ile | 0.403 | 0.398 | **0.40** |
| D3 | Pro-Lys | 0.606 | 0.604 | **0.61** |
| D4 | Pro-Ser | 0.508 | 0.494 | **0.50** |
| D5 | Pro-Trp | 0.768 | 0.788 | **0.78** |
| D6 | Pro-Val | 0.381 | 0.391 | **0.39** |
| D7 | Ser-Asn | 0.623 | 0.604 | **0.61** |
| D8 | Ser-Asp | 0.487 | 0.516 | **0.50** |
| D9 | Ser-Gln | 0.686 | 0.707 | **0.69** |
| D10 | Ser-Glu | 0.516 | 0.548 | **0.53** |
| D11 | Thr-Asp | 0.364 | 0.368 | **0.37** |
| D12 | Thr-Gln | 0.457 | 0.448 | **0.45** |
| E1 | Thr-Phe | 0.29 | 0.28 | **0.29** |
| E2 | Thr-Ser | 0.457 | 0.43 | **0.44** |
| E3 | Trp-Val | 0.358 | 0.357 | **0.36** |
| E4 | Tyr-Ile | 0.405 | 0.423 | **0.41** |
| E5 | Tyr-Val | 0.305 | 0.288 | **0.29** |
| E6 | Val-Ala | 0.385 | 0.385 | **0.39** |
| E7 | Val-Gln | 0.443 | 0.444 | **0.44** |
| E8 | Val-Glu | 0.338 | 0.336 | **0.34** |
| E9 | Val-Lys | 0.468 | 0.478 | **0.47** |
| E10 | Val-Met | 0.295 | 0.266 | **0.28** |
| E11 | Val-Phe | 0.278 | 0.267 | **0.27** |
| E12 | Val-Pro | 0.33 | 0.274 | **0.30** |
| F1 | Val-Ser | 0.451 | 0.429 | **0.44** |
| F2 | β-Ala-Ala | 0.391 | 0.374 | **0.38** |
| F3 | β-Ala-Gly | 0.32 | 0.357 | **0.34** |
| F4 | β-Ala-His | 0.347 | 0.339 | **0.34** |
| F5 | Met-β-His | 0.261 | 0.263 | **0.26** |
| F6 | β-Ala-Phe | 0.24 | 0.269 | **0.26** |
| F7 | D-Ala-D-Ala | 0.184 | 0.189 | **0.19** |
| F8 | D-Ala-Gly | 0.341 | 0.329 | **0.34** |
| F9 | D-Ala-Leu | 0.483 | 0.471 | **0.48** |
| F10 | D-Leu-D-Leu | 0.284 | 0.297 | **0.29** |
| F11 | D-Leu-Gly | 0.169 | 0.179 | **0.17** |
| F12 | D-Leu-Tyr | 0.143 | 0.145 | **0.14** |
| G1 | γ-Glu-Gly | 0.402 | 0.406 | **0.40** |
| G2 | γ-D-Glu-Gly | 0.083 | 0.089 | **0.09** |
| G3 | Gly-D-Ala | 0.309 | 0.278 | **0.29** |
| G4 | Gly-D-Asp | 0.306 | 0.322 | **0.31** |
| G5 | Gly-D-Ser | 0.311 | 0.3 | **0.31** |
| G6 | Gly-D-Thr | 0.181 | 0.168 | **0.18** |
| G7 | Gly-D-Val | 0.133 | 0.127 | **0.13** |
| G8 | Leu-β-Ala | 0.38 | 0.448 | **0.41** |
| G9 | Leu-D-Ala | 0.282 | 0.277 | **0.28** |
| G10 | Phe-β-Ala | 0.266 | 0.258 | **0.26** |
| G11 | Ala-Ala-Ala | 0.427 | 0.432 | **0.43** |
| G12 | D-Ala-Gly-Gly | 0.307 | 0.315 | **0.31** |
| H1 | Gly-Gly-Ala | 0.502 | 0.441 | **0.47** |
| H2 | Gly-Gly-D-Leu | 0.282 | 0.318 | **0.30** |
| H3 | Gly-Gly-Gly | 0.429 | 0.406 | **0.42** |
| H4 | Gly-Gly-Ile | 0.397 | 0.376 | **0.39** |
| H5 | Gly-Gly-Leu | 0.507 | 0.504 | **0.51** |
| H6 | Gly-Gly-Phe | 0.359 | 0.329 | **0.34** |
| H7 | Val-Tyr-Val | 0.186 | 0.175 | **0.18** |
| H8 | Gly-Phe-Phe | 0.388 | 0.486 | **0.44** |
| H9 | Leu-Gly-Gly | 0.361 | 0.359 | **0.36** |
| H10 | Leu-Leu-Leu | 0.34 | 0.239 | **0.29** |
| H11 | Phe-Gly-Gly | 0.291 | 0.239 | **0.27** |
| H12 | Tyr-Gly-Gly | 0.683 | 0.647 | **0.67** |

Table S4. High-throughput DSF screen of McpU^LBD^ with PM6. Peptides that produced a thermal shift ≥ 2.0 were considered positive for interaction.

| Well | Sample | Melting Temperature | ∆Tm | Melting Temperature | ∆Tm | **Average ∆Tm** |
| --- | --- | --- | --- | --- | --- | --- |
| A01 | Negative control | 37.00 |  | 37.50 |  |  |
| A02 | Glutamine | 44.00 |  | 38.50 |  |  |
| A03 | Ala-Ala | 38.50 | 1.5 | 40.00 | 2.5 | **2** |
| A04 | Ala-Arg | 38.50 | 1.5 | 40.00 | 2.5 | **2** |
| A05 | Ala-Asn | 37.50 | 0.5 | 38.00 | 0.5 | **0.5** |
| A06 | Ala-Glu | 37.50 | 0.5 | 37.50 | 0 | **0.25** |
| A07 | Ala-Gly | 38.00 | 1 | 38.00 | 0.5 | **0.75** |
| A08 | Ala-His | 38.50 | 1.5 | 39.00 | 1.5 | **1.5** |
| A09 | Ala-Leu | 41.50 | 4.5 | 39.00 | 1.5 | **3** |
| A10 | Ala-Lys | 42.50 | 5.5 | 40.00 | 2.5 | **4** |
| A11 | Ala-Phe | 42.00 | 5 | 39.50 | 2 | **3.5** |
| A12 | Ala-Pro | 40.00 | 3 | 38.50 | 1 | **2** |
| B01 | Ala-Ser | 37.50 | 0.5 | 39.00 | 1.5 | **1** |
| B02 | Ala-Thr | 43.00 | 6 | 40.50 | 3 | **4.5** |
| B03 | Ala-Trp | 42.50 | 5.5 | 39.00 | 1.5 | **3.5** |
| B04 | Ala-Tyr | 40.50 | 3.5 | 38.00 | 0.5 | **2** |
| B05 | Arg-Ala | 39.00 | 2 | 38.50 | 1 | **1.5** |
| B06 | Arg-Arg | 42.00 | 5 | 39.50 | 2 | **3.5** |
| B07 | Arg-Asp | 37.50 | 0.5 | 37.50 | 0 | **0.25** |
| B08 | Arg-Gln | 37.50 | 0.5 | 37.50 | 0 | **0.25** |
| B09 | Arg-Glu | 38.50 | 1.5 | 37.50 | 0 | **0.75** |
| B10 | Arg-Ile | 38.00 | 1 | 38.00 | 0.5 | **0.75** |
| B11 | Arg-Leu | 39.00 | 2 | 38.00 | 0.5 | **1.25** |
| B12 | Arg-Lys | 39.50 | 2.5 | 38.50 | 1 | **1.75** |
| C01 | Arg-Met | 36.50 | 0 | 38.50 | 1 | **0.5** |
| C02 | Arg-Phe | 40.50 | 3.5 | 38.50 | 1 | **2.25** |
| C03 | Arg-Ser | 39.50 | 2.5 | 38.00 | 0.5 | **1.5** |
| C04 | Arg-Trp | 43.00 | 6 | 39.50 | 2 | **4** |
| C05 | Arg-Tyr | 42.00 | 5 | 39.00 | 1.5 | **3.25** |
| C06 | Arg-Val | 41.50 | 3.5 | 38.00 | 1.5 | **2.5** |
| C07 | Asn-Glu | 38.00 | 1 | 37.50 | 0 | **0.5** |
| C08 | Asn-Val | 39.50 | 2.5 | 37.50 | 0 | **1.25** |
| C09 | Asp-Asp | 38.50 | 1.5 | 37.50 | 0 | **0.75** |
| C10 | Asp-Glu | 36.00 | 0 | 37.50 | 0 | **0** |
| C11 | Asp-Leu | 41.00 | 5 | 38.00 | 0.5 | **2.75** |
| C12 | Asp-Lys | 40.00 | 3 | 39.00 | 1.5 | **2.25** |
| D01 | Asp-Phe | 39.50 | 2.5 | 37.50 | 0 | **1.25** |
| D02 | Asp-Trp | 40.50 | 3.5 | 38.00 | 0.5 | **2** |
| D03 | Asp-Val | 37.00 | 0 | 37.50 | 0 | **0** |
| D04 | Cys-Gly | 39.50 | 2.5 | 37.50 | 0 | **1.25** |
| D05 | Gln-Gln | 38.00 | 1 | 37.00 | 0 | **0.5** |
| D06 | Gln-Gly | 37.50 | 0.5 | 37.00 | 0 | **0.25** |
| D07 | Glu-Asp | 38.00 | 1 | 37.50 | 0 | **0.5** |
| D08 | Glu-Glu | 41.00 | 4 | 37.00 | 0 | **2** |
| D09 | Glu-Gly | 39.00 | 2 | 37.00 | 0 | **1** |
| D10 | Glu-Ser | 37.50 | 0.5 | 37.50 | 0 | **0.25** |
| D11 | Glu-Trp | 40.00 | 3 | 38.00 | 0.5 | **1.75** |
| D12 | Glu-Tyr | 40.50 | 3.5 | 39.00 | 1.5 | **2.5** |
| E01 | Glu-Val | 39.00 | 2 | 38.00 | 0.5 | **1.25** |
| E02 | Gly-Ala | 40.00 | 3 | 38.00 | 0.5 | **1.75** |
| E03 | Gly-Arg | 40.50 | 3.5 | 37.50 | 0 | **1.75** |
| E04 | Gly-Cys | 37.00 | 0 | 37.00 | 0 | **0** |
| E05 | Gly-Gly | 36.50 | 0 | 37.50 | 0 | **0** |
| E06 | Gly-His | 37.00 | 0 | 37.00 | 0 | **0** |
| E07 | Gly-Leu | 38.50 | 1.5 | 37.50 | 0 | **0.75** |
| E08 | Gly-Lys | 42.50 | 3.5 | 38.50 | 1 | **2.25** |
| E09 | Gly-Met | 39.50 | 2.5 | 37.50 | 0 | **1.25** |
| E10 | Gly-Phe | 38.00 | 1 | 37.50 | 0 | **0.5** |
| E11 | Gly-Pro | 38.50 | 1.5 | 38.00 | 0.5 | **1** |
| E12 | Gly-Ser | 38.50 | 1.5 | 37.50 | 0 | **0.75** |
| F01 | Gly-Thr | 38.50 | 1.5 | 37.50 | 0 | **0.75** |
| F02 | Gly-Trp | 37.00 | 0 | 38.00 | 0.5 | **0.25** |
| F03 | Gly-Tyr | 39.50 | 2.5 | 38.00 | 0.5 | **1.5** |
| F04 | Gly-Val | 37.50 | 0.5 | 37.00 | 0 | **0.25** |
| F05 | His-Asp | 39.00 | 2 | 37.50 | 0 | **1** |
| F06 | His-Gly | 37.50 | 0.5 | 37.50 | 0 | **0.25** |
| F07 | His-Leu | 40.00 | 3 | 37.50 | 0 | **1.5** |
| F08 | His-Lys | 36.00 | 0 | 37.50 | 0 | **0** |
| F09 | His-Met | 38.50 | 1.5 | 37.50 | 0 | **0.75** |
| F10 | His-Pro | 40.00 | 3 | 39.00 | 1.5 | **2.25** |
| F11 | His-Ser | 37.50 | 0.5 | 37.50 | 0 | **0.25** |
| F12 | His-Trp | 36.50 | 0 | 38.00 | 0.5 | **0.25** |
| G01 | His-Tyr | 38.50 | 1.5 | 37.50 | 0 | **0.75** |
| G02 | His-Val | 41.00 | 4 | 39.00 | 1.5 | **2.75** |
| G03 | Ile-Ala | 37.50 | 0.5 | 39.00 | 1.5 | **1** |
| G04 | Ile-Arg | 41.50 | 3.5 | 38.50 | 2 | **2.75** |
| G05 | Ile-Gln | 38.00 | 1 | 37.50 | 0 | **0.5** |
| G06 | Ile-Gly | 39.50 | 2.5 | 37.00 | 0 | **1.25** |
| G07 | Ile-His | 40.50 | 3.5 | 37.50 | 0 | **1.75** |
| G08 | Ile-Ile | 40.50 | 3.5 | 39.50 | 2 | **2.75** |
| G09 | Ile-Met | 40.50 | 2.5 | 38.50 | 2 | **2.25** |
| G10 | Ile-Phe | 40.00 | 3 | 39.00 | 1.5 | **2.25** |
| G11 | Ile-Pro | 39.50 | 2.5 | 38.50 | 1 | **1.75** |
| G12 | Ile-Ser | 41.00 | 4 | 38.50 | 1 | **2.5** |
| H01 | Ile-Trp | 40.50 | 3.5 | 39.00 | 1.5 | **2.5** |
| H02 | Ile-Tyr | 40.50 | 3.5 | 38.50 | 1 | **2.25** |
| H03 | Ile-Val | 41.00 | 4 | 39.50 | 2 | **3** |
| H04 | Leu-Ala | 43.00 | 6 | 40.50 | 3 | **4.5** |
| H05 | Leu-Arg | 42.00 | 5 | 38.00 | 0.5 | **2.75** |
| H06 | Leu-Asp | 40.00 | 3 | 38.00 | 0.5 | **1.75** |
| H07 | Leu-Glu | 41.50 | 4.5 | 38.00 | 0.5 | **2.5** |
| H08 | Leu-Gly | 34.00 | 0 | 38.00 | 0.5 | **0.25** |
| H09 | Leu-Ile | 40.00 | 3 | 38.50 | 1 | **2** |
| H10 | Leu-Leu | 40.50 | 3.5 | 39.00 | 1.5 | **2.5** |
| H11 | Leu-Met | 41.50 | 4.5 | 39.00 | 1.5 | **3** |
| H12 | Leu-Phe | 40.50 | 3.5 | 38.50 | 1 | **2.25** |

Table S5. High-throughput DSF screen of McpU^LBD^ with PM7. Peptides that produced a thermal shift ≥ 2.0 were considered positive for interaction.

| Well | Sample | Melting Temperature | ∆Tm | Melting Temperature | ∆Tm | **Average ∆Tm** |
| --- | --- | --- | --- | --- | --- | --- |
| A01 | Negative control | 37 |  | 39 |  |  |
| A02 | Glutamine | 43.5 |  | 44 |  |  |
| A03 | Leu-Ser | 38.5 | 1.5 | 41.5 | 2.5 | **2** |
| A04 | Leu-Trp | 37.5 | 0.5 | 39.5 | 0.5 | **0.5** |
| A05 | Leu-Val | 38.5 | 1.5 | 42.5 | 3.5 | **2.5** |
| A06 | Lys-Ala | 39.5 | 2.5 | 41 | 2 | **2.25** |
| A07 | Lys-Arg | 38.5 | 1.5 | 41.5 | 2.5 | **2** |
| A08 | Lys-Glu | 37 | 0 | 39.5 | 0.5 | **0.25** |
| A09 | Lys-Ile | 39.5 | 2.5 | 42.5 | 3.5 | **3** |
| A10 | Lys-Leu | 39.5 | 2.5 | 44 | 5 | **2.5** |
| A11 | Lys-Lys | 37.5 | 0.5 | 41 | 2 | **0.8** |
| A12 | Lys-Phe | 38 | 1 | 42.5 | 3.5 | **2.25** |
| B1 | Lys-Pro | 37.5 | 0.5 | 40 | 1 | **0.8** |
| B2 | Lys-Ser | 39 | 2 | 43 | 4 | **3** |
| B3 | Lys-Thr | 38.5 | 1.5 | 41.5 | 2.5 | **2** |
| B4 | Lys-Trp | 38 | 1 | 41 | 2 | **1.5** |
| B5 | Lys-Tyr | 38 | 1 | 42.5 | 3.5 | **2.25** |
| B6 | Lys-Val | 40.5 | 3.5 | 42.5 | 3.5 | **3.5** |
| B7 | Met-Arg | 37 | 0 | 40.5 | 1.5 | **0.8** |
| B8 | Met-Asp | 37 | 0 | 40 | 1 | **0.5** |
| B9 | Met-Gln | 37.5 | 0.5 | 42.5 | 3.5 | **2** |
| B10 | Met-Glu | 37.5 | 0.5 | 42.5 | 3.5 | **2** |
| B11 | Met-Gly | 37 | 0 | 40.5 | 1.5 | **0.8** |
| B12 | Met-His | 37.5 | 0.5 | 42 | 3 | **1.75** |
| C1 | Met-Ile | 38 | 1 | 42.5 | 3.5 | **2.25** |
| C2 | Met-Leu | 38.5 | 1.5 | 43 | 4 | **2.75** |
| C3 | Met-Lys | 37.5 | 0.5 | 42 | 3 | **1.75** |
| C4 | Met-Met | 38.5 | 1.5 | 42 | 3 | **2.25** |
| C5 | Met-Phe | 37.5 | 0.5 | 42.5 | 3.5 | **2** |
| C6 | Met-Pro | 39 | 2 | 41 | 2 | **2** |
| C7 | Met-Trp | 39.5 | 2.5 | 42 | 3 | **2.75** |
| C8 | Met-Val | 38.5 | 1.5 | 43.5 | 4.5 | **3** |
| C9 | Phe-Ala | 38.5 | 1.5 | 42.5 | 3.5 | **2.5** |
| C10 | Phe-Gly | 36 | 0 | 41 | 2 | **1** |
| C11 | Phe-Ile | 38.5 | 1.5 | 41.5 | 2.5 | **2** |
| C12 | Phe-Phe | 36 | 0 | 39 | 0 | **0** |
| D1 | Phe-Pro | 35.5 | 0 | 40 | 1 | **0.5** |
| D2 | Phe-Ser | 37.5 | 0.5 | 42.5 | 3.5 | **2** |
| D3 | Phe-Trp | 39.5 | 2.5 | 44 | 5 | **3.25** |
| D4 | Pro-Ala | 38 | 1 | 39.5 | 0.5 | **0.75** |
| D5 | Pro-Asp | 37 | 0 | 39 | 0 | **0** |
| D6 | Pro-Gln | 37 | 0 | 41 | 2 | **1** |
| D7 | Pro-Gly | 37.5 | 0.5 | 39.5 | 0.5 | **0.5** |
| D8 | Pro-Hyp | 36 | 0 | 40 | 1 | **0.5** |
| D9 | Pro-Leu | 37 | 0 | 40 | 1 | **0.5** |
| D10 | Pro-Phe | 37.5 | 0.5 | 40 | 1 | **0.75** |
| D11 | Pro-Pro | 38 | 1 | 42 | 3 | **2** |
| D12 | Pro-Tyr | 36.5 | 0 | 40 | 1 | **0.5** |
| E1 | Ser-Ala | 39 | 2 | 43.5 | 4.5 | **3.25** |
| E2 | Ser-Gly | 36 | 0 | 40.5 | 1.5 | **0.75** |
| E3 | Ser-His | 36 | 0 | 41 | 2 | **1** |
| E4 | Ser-Leu | 35.5 | 0 | 41 | 2 | **1** |
| E5 | Ser-Met | 38.5 | 1.5 | 43 | 4 | **2.75** |
| E6 | Ser-Phe | 37.5 | 0.5 | 42 | 3 | **1.75** |
| E7 | Ser-Pro | 36 | 0 | 40 | 1 | **0.5** |
| E8 | Ser-Ser | 37.5 | 0.5 | 42.5 | 3.5 | **2** |
| E9 | Ser-Tyr | 39.5 | 2.5 | 42 | 3 | **2.75** |
| E10 | Ser-Val | 38 | 1 | 42.5 | 3.5 | **2.25** |
| E11 | Thr-Ala | 37.5 | 0.5 | 41 | 2 | **1.25** |
| E12 | Thr-Arg | 37.5 | 0.5 | 41 | 2 | **1.25** |
| F1 | Thr-Glu | 36 | 0 | 40.5 | 1.5 | **0.75** |
| F2 | Thr-Gly | 36.5 | 0 | 40 | 1 | **0.5** |
| F3 | Thr-Leu | 37.5 | 0.5 | 41.5 | 2.5 | **1.5** |
| F4 | Thr-Met | 37.5 | 0.5 | 41 | 2 | **1.25** |
| F5 | Thr-Pro | 35.5 | 0 | 40 | 1 | **0.5** |
| F6 | Trp-Ala | 39 | 2 | 41.5 | 2.5 | **2.25** |
| F7 | Trp-Arg | 38.5 | 1.5 | 42.5 | 3.5 | **2.5** |
| F8 | Trp-Asp | 37.5 | 0.5 | 41 | 2 | **1.5** |
| F9 | Trp-Glu | 37 | 0 | 40.5 | 1.5 | **0.75** |
| F10 | Trp-Gly | 37 | 0 | 41.5 | 2.5 | **1.25** |
| F11 | Trp-Leu | 38 | 1 | 41.5 | 2.5 | **1.75** |
| F12 | Trp-Lys | 39.5 | 2.5 | 43 | 4 | **3.25** |
| G1 | Trp-Phe | 37 | 0 | 39.5 | 0.5 | **0.25** |
| G2 | Trp-Ser | 37.5 | 0.5 | 42 | 3 | **1.75** |
| G3 | Trp-Trp | 37.5 | 0.5 | 42 | 3 | **1.75** |
| G4 | Trp-Tyr | 37.5 | 0.5 | 41 | 2 | **1.25** |
| G5 | Tyr-Ala | 37.5 | 0.5 | 42 | 3 | **1.75** |
| G6 | Tyr-Gln | 36 | 0 | 41.5 | 2.5 | **1.25** |
| G7 | Tyr-Glu | 35.5 | 0 | 41.5 | 2.5 | **1.25** |
| G8 | Tyr-Gly | 35.5 | 0 | 41 | 2 | **1** |
| G9 | Tyr-His | 35.5 | 0 | 41 | 2 | **1** |
| G10 | Tyr-Leu | 38 | 1 | 42 | 3 | **2** |
| G11 | Tyr-Lys | 37.5 | 0.5 | 42.5 | 3.5 | **2** |
| G12 | Tyr-Phe | 38.5 | 1.5 | 42 | 3 | **2.25** |
| H1 | Tyr-Trp | 39.5 | 2.5 | 40.5 | 1.5 | **2** |
| H2 | Tyr-Tyr | 38.5 | 1.5 | 40.5 | 1.5 | **1.5** |
| H3 | Val-Arg | 37.5 | 0.5 | 42.5 | 3.5 | **2** |
| H4 | Val-Asn | 37.5 | 0.5 | 41 | 2 | **1.25** |
| H5 | Val-Asp | 36.5 | 0 | 39 | 0 | **0** |
| H6 | Val-Gly | 36.5 | 0 | 40.5 | 1.5 | **0.75** |
| H7 | Val-His | 38.5 | 1.5 | 42 | 3 | **2.25** |
| H8 | Val-Ile | 39.5 | 2.5 | 44 | 5 | **3.75** |
| H9 | Val-Leu | 37.5 | 0.5 | 43 | 4 | **2.25** |
| H10 | Val-Tyr | 38 | 1 | 42 | 3 | **2** |
| H11 | Val-Val | 37.5 | 0.5 | 42.5 | 3.5 | **2** |
| H12 | γ-Glu-Gly | 37.5 | 0.5 | 40 | 1 | **0.75** |

Table S6. High-throughput DSF screen of McpU^LBD^ with PM8. Peptides that produced a thermal shift ≥ 2.0 were considered positive for interaction.

| Well | Sample | Melting Temperature | ∆Tm | Melting Temperature | ∆Tm | **Average ∆Tm** |
| --- | --- | --- | --- | --- | --- | --- |
| A01 | Negative control | 38.00 |  | 41.50 |  |  |
| A02 | Glutamine | 45.00 |  | 44.00 |  |  |
| A03 | Ala-Asp | 39.00 | 1 | 44.00 | 2.5 | **1.75** |
| A04 | Ala-Gln | 41.50 | 3.5 | 47.00 | 5.5 | **4.5** |
| A05 | Ala-Ile | 40.00 | 2 | 46.50 | 5 | **3.5** |
| A06 | Ala-Met | 42.00 | 4 | 46.50 | 5 | **4.5** |
| A07 | Ala-Val | 42.50 | 4.5 | 47.00 | 5.5 | **5** |
| A08 | Asp-Ala | 38.00 | 0 | 43.50 | 2 | **1** |
| A09 | Asp-Gln | 40.50 | 2.5 | 46.50 | 5 | **3.75** |
| A10 | Asp-Gly | 38.00 | 0 | 41.50 | 0 | **0** |
| A11 | Glu-Ala | 38.50 | 0.5 | 41.50 | 0 | **0.25** |
| A12 | Gly-Asn | 41.50 | 3.5 | 43.50 | 2 | **2.75** |
| B1 | Gly-Asp | 38.00 | 0 | 41.50 | 0 | **0** |
| B2 | Gly-Ile | 36.50 | 0 | 43.00 | 1.5 | **0.75** |
| B3 | His-Ala | 42.50 | 4.5 | 48.00 | 6.5 | **5.5** |
| B4 | His-Glu | 39.50 | 1.5 | 47.50 | 6 | **3.75** |
| B5 | His-His | 38.00 | 0 | 41.50 | 0 | **0** |
| B6 | Ile-Asn | 37.00 | 0 | 43.00 | 1.5 | **0.75** |
| B7 | Ile-Leu | 38.50 | 0.5 | 44.00 | 2.5 | **1.5** |
| B8 | Leu-Asn | 39.50 | 1.5 | 44.50 | 3 | **2.25** |
| B9 | Leu-His | 38.00 | 0 | 43.50 | 2 | **1** |
| B10 | Leu-Pro | 37.00 | 0 | 42.50 | 1 | **0.5** |
| B11 | Leu-Tyr | 40.50 | 2.5 | 46.00 | 4.5 | **3.5** |
| B12 | Lys-Asp | 39.00 | 1 | 44.50 | 3 | **2** |
| C1 | Lys-Gly | 38.50 | 0.5 | 44.50 | 3 | **1.75** |
| C2 | Lys-Met | 40.00 | 2 | 45.00 | 3.5 | **2.75** |
| C3 | Met-Thr | 41.50 | 3.5 | 46.50 | 5 | **4.25** |
| C4 | Met-Tyr | 37.50 | 0 | 41.50 | 0 | **0** |
| C5 | Phe-Asp | 37.50 | 0 | 43.00 | 1.5 | **0.75** |
| C6 | Phe-Glu | 38.00 | 0 | 43.50 | 2 | **1** |
| C7 | Gln-Glu | 37.00 | 0 | 41.50 | 0 | **0** |
| C8 | Phe-Met | 37.00 | 0 | 41.50 | 0 | **0** |
| C9 | Phe-Tyr | 37.50 | 0 | 41.50 | 0 | **0** |
| C10 | Phe-Val | 40.50 | 2.5 | 45.00 | 3.5 | **3** |
| C11 | Pro-Arg | 37.50 | 0 | 41.50 | 0 | **0** |
| C12 | Pro-Asn | 38.00 | 0 | 42.00 | 0.5 | **0.25** |
| D1 | Pro-Glu | 38.00 | 0 | 42.50 | 1 | **0.5** |
| D2 | Pro-Ile | 42.00 | 4 | 47.50 | 6 | **5** |
| D3 | Pro-Lys | 37.50 | 0 | 43.00 | 1.5 | **0.75** |
| D4 | Pro-Ser | 38.50 | 0.5 | 43.00 | 1.5 | **1** |
| D5 | Pro-Trp | 38.50 | 0.5 | 43.00 | 1.5 | **1** |
| D6 | Pro-Val | 36.50 | 0 | 41.50 | 0 | **0** |
| D7 | Ser-Asn | 35.50 | 0 | 42.50 | 1 | **0.5** |
| D8 | Ser-Asp | 37.00 | 0 | 41.50 | 0 | **0** |
| D9 | Ser-Gln | 37.50 | 0 | 43.50 | 2 | **1** |
| D10 | Ser-Glu | 37.00 | 0 | 42.50 | 1 | **0.5** |
| D11 | Thr-Asp | 37.50 | 0 | 42.00 | 0.5 | **0.25** |
| D12 | Thr-Gln | 38.00 | 0 | 42.50 | 1 | **0.5** |
| E1 | Thr-Phe | 38.50 | 0.5 | 41.50 | 0 | **0.25** |
| E2 | Thr-Ser | 37.00 | 0 | 43.00 | 1.5 | **0.75** |
| E3 | Trp-Val | 40.00 | 2 | 44.50 | 3 | **2.5** |
| E4 | Tyr-Ile | 38.00 | 0 | 47.50 | 6 | **3** |
| E5 | Tyr-Val | 36.50 | 0 | 41.50 | 0 | **0** |
| E6 | Val-Ala | 41.50 | 3.5 | 46.50 | 5 | **4.25** |
| E7 | Val-Gln | 38.50 | 0.5 | 44.50 | 3 | **1.75** |
| E8 | Val-Glu | 36.50 | 0 | 43.50 | 2 | **1** |
| E9 | Val-Lys | 39.50 | 1.5 | 45.00 | 3.5 | **2.5** |
| E10 | Val-Met | 40.50 | 2.5 | 46.00 | 4.5 | **3.5** |
| E11 | Val-Phe | 44.50 | 6.5 | 45.00 | 3.5 | **5** |
| E12 | Val-Pro | 42.50 | 4.5 | 44.50 | 3 | **3.75** |
| F1 | Val-Ser | 40.00 | 2 | 45.00 | 3.5 | **2.75** |
| F2 | β-Ala-Ala | 37.50 | 0 | 42.00 | 0.5 | **0.25** |
| F3 | β-Ala-Gly | 37.50 | 0 | 41.50 | 0 | **0** |
| F4 | β-Ala-His | 38.00 | 0 | 42.00 | 1 | **0.5** |
| F5 | Met-β-His | 37.00 | 0 | 41.50 | 0 | **0** |
| F6 | β-Ala-Phe | 37.00 | 0 | 42.50 | 1 | **0.5** |
| F7 | D-Ala-D-Ala | 37.00 | 0 | 41.50 | 0 | **0** |
| F8 | D-Ala-Gly | 37.00 | 0 | 41.50 | 0 | **0** |
| F9 | D-Ala-Leu | 37.50 | 0 | 42.00 | 0.5 | **0.25** |
| F10 | D-Leu-D-Leu | 37.50 | 0 | 41.50 | 0 | **0** |
| F11 | D-Leu-Gly | 38.00 | 0 | 41.50 | 0 | **0** |
| F12 | D-Leu-Tyr | 38.00 | 0 | 43.50 | 2 | **1** |
| G1 | γ-Glu-Gly | 37.50 | 0 | 42.00 | 0.5 | **0.25** |
| G2 | γ-D-Glu-Gly | 38.00 | 0 | 42.00 | 0.5 | **0.25** |
| G3 | Gly-D-Ala | 37.00 | 0 | 41.50 | 0 | **0** |
| G4 | Gly-D-Asp | 38.00 | 0 | 42.00 | 0.5 | **0.25** |
| G5 | Gly-D-Ser | 38.00 | 0 | 41.50 | 0 | **0** |
| G6 | Gly-D-Thr | 37.50 | 0 | 42.00 | 0.5 | **0.25** |
| G7 | Gly-D-Val | 37.50 | 0 | 42.00 | 0.5 | **0.25** |
| G8 | Leu-β-Ala | 38.00 | 0 | 41.50 | 0 | **0** |
| G9 | Leu-D-Ala | 37.50 | 0 | 41.50 | 0 | **0** |
| G10 | Phe-β-Ala | 36.50 | 0 | 41.50 | 0 | **0** |
| G11 | Ala-Ala-Ala | 39.00 | 1 | 44.00 | 2.5 | **1.75** |
| G12 | D-Ala-Gly-Gly | 38.00 | 0 | 41.50 | 0 | **0** |
| H1 | Gly-Gly-Ala | 39.50 | 1.5 | 41.50 | 0 | **0.75** |
| H2 | Gly-Gly-D-Leu | 38.50 | 0.5 | 42.00 | 0.5 | **0.5** |
| H3 | Gly-Gly-Gly | 38.50 | 0.5 | 41.50 | 0 | **0.25** |
| H4 | Gly-Gly-Ile | 38.00 | 0 | 41.50 | 0 | **0** |
| H5 | Gly-Gly-Leu | 38.50 | 0.5 | 42.00 | 0.5 | **0.5** |
| H6 | Gly-Gly-Phe | 39.50 | 1.5 | 41.50 | 0 | **0.75** |
| H7 | Val-Tyr-Val | 38.00 | 0 | 41.50 | 0 | **0** |
| H8 | Gly-Phe-Phe | 38.50 | 0.5 | 41.50 | 0 | **0.25** |
| H9 | Leu-Gly-Gly | 38.00 | 0 | 42.50 | 1 | **0.5** |
| H10 | Leu-Leu-Leu | 39.00 | 1 | 41.50 | 0 | **0.5** |
| H11 | Phe-Gly-Gly | 38.50 | 0.5 | 42.00 | 0.5 | **0.5** |
| H12 | Tyr-Gly-Gly | 39.00 | 1 | 42.00 | 0.5 | **0.75** |

^[1]^

Table S7. Biolog growth assay of ∆*dppA1dppA2* with phenotypic microarray plates 6 (PM6 peptide nitrogen sources). Peptides in wells with an average absorbance (590 nm) ≥ 0.2 were considered positive for utilization.

| Well | Sample | Absorbance (590) 1 | Absorbance (590) 2 | Average  Absorbance |
| --- | --- | --- | --- | --- |
| A01 | Negative control | 0.13 | 0.117 | **0.12** |
| A02 | Positive control | 0.312 | 0.315 | **0.31** |
| A03 | Ala-Ala | 0.339 | 0.352 | **0.35** |
| A04 | Ala-Arg | 0.215 | 0.225 | **0.22** |
| A05 | Ala-Asn | 0.288 | 0.294 | **0.29** |
| A06 | Ala-Glu | 0.303 | 0.311 | **0.31** |
| A07 | Ala-Gly | 0.279 | 0.293 | **0.29** |
| A08 | Ala-His | 0.305 | 0.317 | **0.31** |
| A09 | Ala-Leu | 0.32 | 0.375 | **0.35** |
| A10 | Ala-Lys | 0.317 | 0.347 | **0.33** |
| A11 | Ala-Phe | 0.235 | 0.258 | **0.25** |
| A12 | Ala-Pro | 0.302 | 0.325 | **0.31** |
| B1 | Ala-Ser | 0.27 | 0.196 | **0.23** |
| B2 | Ala-Thr | 0.2 | 0.258 | **0.23** |
| B3 | Ala-Trp | 0.277 | 0.27 | **0.27** |
| B4 | Ala-Tyr | 0.297 | 0.274 | **0.29** |
| B5 | Arg-Ala | 0.298 | 0.272 | **0.29** |
| B6 | Arg-Arg | 0.237 | 0.242 | **0.24** |
| B7 | Arg-Asp | 0.273 | 0.258 | **0.27** |
| B8 | Arg-Gln | 0.312 | 0.293 | **0.30** |
| B9 | Arg-Glu | 0.287 | 0.272 | **0.28** |
| B10 | Arg-Ile | 0.246 | 0.225 | **0.24** |
| B11 | Arg-Leu | 0.314 | 0.308 | **0.31** |
| B12 | Arg-Lys | 0.287 | 0.317 | **0.30** |
| C1 | Arg-Met | 0.197 | 0.138 | **0.17** |
| C2 | Arg-Phe | 0.213 | 0.162 | **0.19** |
| C3 | Arg-Ser | 0.248 | 0.209 | **0.23** |
| C4 | Arg-Trp | 0.248 | 0.232 | **0.24** |
| C5 | Arg-Tyr | 0.318 | 0.285 | **0.30** |
| C6 | Arg-Val | 0.213 | 0.186 | **0.19** |
| C7 | Asn-Glu | 0.26 | 0.227 | **0.24** |
| C8 | Asn-Val | 0.226 | 0.199 | **0.21** |
| C9 | Asp-Asp | 0.194 | 0.172 | **0.18** |
| C10 | Asp-Glu | 0.219 | 0.197 | **0.21** |
| C11 | Asp-Leu | 0.258 | 0.23 | **0.24** |
| C12 | Asp-Lys | 0.337 | 0.293 | **0.32** |
| D1 | Asp-Phe | 0.242 | 0.165 | **0.20** |
| D2 | Asp-Trp | 0.183 | 0.174 | **0.18** |
| D3 | Asp-Val | 0.156 | 0.152 | **0.15** |
| D4 | Cys-Gly | 0.175 | 0.157 | **0.17** |
| D5 | Gln-Gln | 0.233 | 0.25 | **0.24** |
| D6 | Gln-Gly | 0.274 | 0.238 | **0.26** |
| D7 | Glu-Asp | 0.145 | 0.136 | **0.14** |
| D8 | Glu-Glu | 0.183 | 0.171 | **0.18** |
| D9 | Glu-Gly | 0.225 | 0.199 | **0.21** |
| D10 | Glu-Ser | 0.294 | 0.256 | **0.28** |
| D11 | Glu-Trp | 0.305 | 0.302 | **0.30** |
| D12 | Glu-Tyr | 0.306 | 0.279 | **0.29** |
| E1 | Glu-Val | 0.206 | 0.152 | **0.18** |
| E2 | Gly-Ala | 0.178 | 0.163 | **0.17** |
| E3 | Gly-Arg | 0.246 | 0.193 | **0.22** |
| E4 | Gly-Cys | 0.641 | 0.633 | **0.64** |
| E5 | Gly-Gly | 0.186 | 0.183 | **0.19** |
| E6 | Gly-His | 0.252 | 0.215 | **0.23** |
| E7 | Gly-Leu | 0.275 | 0.228 | **0.25** |
| E8 | Gly-Lys | 0.22 | 0.222 | **0.22** |
| E9 | Gly-Met | 0.455 | 0.423 | **0.44** |
| E10 | Gly-Phe | 0.242 | 0.213 | **0.23** |
| E11 | Gly-Pro | 0.236 | 0.213 | **0.23** |
| E12 | Gly-Ser | 0.288 | 0.256 | **0.27** |
| F1 | Gly-Thr | 0.132 | 0.128 | **0.13** |
| F2 | Gly-Trp | 0.228 | 0.192 | **0.21** |
| F3 | Gly-Tyr | 0.255 | 0.226 | **0.24** |
| F4 | Gly-Val | 0.188 | 0.158 | **0.17** |
| F5 | His-Asp | 0.24 | 0.204 | **0.22** |
| F6 | His-Gly | 0.222 | 0.192 | **0.21** |
| F7 | His-Leu | 0.215 | 0.182 | **0.19** |
| F8 | His-Lys | 0.254 | 0.22 | **0.24** |
| F9 | His-Met | 0.357 | 0.318 | **0.34** |
| F10 | His-Pro | 0.211 | 0.203 | **0.21** |
| F11 | His-Ser | 0.197 | 0.184 | **0.19** |
| F12 | His-Trp | 0.62 | 0.601 | **0.61** |
| G1 | His-Tyr | 0.294 | 0.25 | **0.27** |
| G2 | His-Val | 0.23 | 0.164 | **0.19** |
| G3 | Ile-Ala | 0.163 | 0.134 | **0.15** |
| G4 | Ile-Arg | 0.159 | 0.157 | **0.16** |
| G5 | Ile-Gln | 0.16 | 0.133 | **0.15** |
| G6 | Ile-Gly | 0.17 | 0.145 | **0.16** |
| G7 | Ile-His | 0.243 | 0.199 | **0.22** |
| G8 | Ile-Ile | 0.125 | 0.12 | **0.12** |
| G9 | Ile-Met | 0.306 | 0.248 | **0.28** |
| G10 | Ile-Phe | 0.175 | 0.147 | **0.16** |
| G11 | Ile-Pro | 0.164 | 0.165 | **0.167** |
| G12 | Ile-Ser | 0.255 | 0.224 | **0.24** |
| H1 | Ile-Trp | 0.277 | 0.27 | **0.27** |
| H2 | Ile-Tyr | 0.453 | 0.386 | **0.42** |
| H3 | Ile-Val | 0.165 | 0.138 | **0.15** |
| H4 | Leu-Ala | 0.273 | 0.183 | **0.23** |
| H5 | Leu-Arg | 0.215 | 0.223 | **0.22** |
| H6 | Leu-Asp | 0.244 | 0.125 | **0.19** |
| H7 | Leu-Glu | 0.212 | 0.116 | **0.16** |
| H8 | Leu-Gly | 0.194 | 0.155 | **0.18** |
| H9 | Leu-Ile | 0.165 | 0.158 | **0.16** |
| H10 | Leu-Leu | 0.181 | 0.176 | **0.18** |
| H11 | Leu-Met | 0.22 | 0.132 | **0.18** |
| H12 | Leu-Phe | 0.277 | 0.172 | **0.23** |

Table S8. Biolog growth assay of ∆*dppA1dppA2* with phenotypic microarray plates 7 (PM7 peptide nitrogen sources). Peptides in wells with an average absorbance (590 nm) ≥ 0.2 were considered positive for utilization.

| Well | Sample | Absorbance (590) 1 | Absorbance (590) 2 | Average  Absorbance |
| --- | --- | --- | --- | --- |
| A01 | Negative control | 0.166 | 0.165 | **0.17** |
| A02 | Positive control | 0.308 | 0.305 | **0.31** |
| A03 | Leu-Ser | 0.25 | 0.245 | **0.25** |
| A04 | Leu-Trp | 0.276 | 0.274 | **0.28** |
| A05 | Leu-Val | 0.195 | 0.2 | **0.19** |
| A06 | Lys-Ala | 0.265 | 0.261 | **0.26** |
| A07 | Lys-Arg | 0.249 | 0.242 | **0.25** |
| A08 | Lys-Glu | 0.22 | 0.214 | **0.22** |
| A09 | Lys-Ile | 0.18 | 0.176 | **0.18** |
| A10 | Lys-Leu | 0.241 | 0.241 | **0.24** |
| A11 | Lys-Lys | 0.383 | 0.363 | **0.37** |
| A12 | Lys-Phe | 0.375 | 0.374 | **0.38** |
| B1 | Lys-Pro | 0.172 | 0.165 | **0.17** |
| B2 | Lys-Ser | 0.219 | 0.219 | **0.22** |
| B3 | Lys-Thr | 0.188 | 0.187 | **0.19** |
| B4 | Lys-Trp | 0.222 | 0.217 | **0.22** |
| B5 | Lys-Tyr | 0.236 | 0.234 | **0.24** |
| B6 | Lys-Val | 0.199 | 0.202 | **0.20** |
| B7 | Met-Arg | 0.326 | 0.315 | **0.32** |
| B8 | Met-Asp | 0.114 | 0.12 | **0.12** |
| B9 | Met-Gln | 0.174 | 0.18 | **0.18** |
| B10 | Met-Glu | 0.324 | 0.326 | **0.33** |
| B11 | Met-Gly | 0.282 | 0.283 | **0.28** |
| B12 | Met-His | 0.323 | 0.322 | **0.32** |
| C1 | Met-Ile | 0.137 | 0.137 | **0.14** |
| C2 | Met-Leu | 0.135 | 0.143 | **0.14** |
| C3 | Met-Lys | 0.162 | 0.166 | **0.16** |
| C4 | Met-Met | 0.12 | 0.118 | **0.12** |
| C5 | Met-Phe | 0.125 | 0.129 | **0.13** |
| C6 | Met-Pro | 0.159 | 0.161 | **0.16** |
| C7 | Met-Trp | 0.199 | 0.205 | **0.20** |
| C8 | Met-Val | 0.166 | 0.164 | **0.17** |
| C9 | Phe-Ala | 0.222 | 0.218 | **0.22** |
| C10 | Phe-Gly | 0.244 | 0.238 | **0.24** |
| C11 | Phe-Ile | 0.352 | 0.342 | **0.35** |
| C12 | Phe-Phe | 0.157 | 0.152 | **0.16** |
| D1 | Phe-Pro | 0.143 | 0.145 | **0.14** |
| D2 | Phe-Ser | 0.18 | 0.179 | **0.18** |
| D3 | Phe-Trp | 0.128 | 0.123 | **0.13** |
| D4 | Pro-Ala | 0.204 | 0.207 | **0.21** |
| D5 | Pro-Asp | 0.276 | 0.273 | **0.28** |
| D6 | Pro-Gln | 0.28 | 0.282 | **0.28** |
| D7 | Pro-Gly | 0.264 | 0.254 | **0.26** |
| D8 | Pro-Hyp | 0.254 | 0.256 | **0.26** |
| D9 | Pro-Leu | 0.328 | 0.33 | **0.33** |
| D10 | Pro-Phe | 0.264 | 0.262 | **0.26** |
| D11 | Pro-Pro | 0.35 | 0.349 | **0.35** |
| D12 | Pro-Tyr | 0.421 | 0.406 | **0.41** |
| E1 | Ser-Ala | 0.402 | 0.369 | **0.39** |
| E2 | Ser-Gly | 0.18 | 0.187 | **0.18** |
| E3 | Ser-His | 0.346 | 0.346 | **0.35** |
| E4 | Ser-Leu | 0.235 | 0.244 | **0.24** |
| E5 | Ser-Met | 0.19 | 0.186 | **0.19** |
| E6 | Ser-Phe | 0.198 | 0.199 | **0.19** |
| E7 | Ser-Pro | 0.238 | 0.239 | **0.24** |
| E8 | Ser-Ser | 0.374 | 0.376 | **0.38** |
| E9 | Ser-Tyr | 0.307 | 0.321 | **0.31** |
| E10 | Ser-Val | 0.312 | 0.312 | **0.31** |
| E11 | Thr-Ala | 0.246 | 0.246 | **0.25** |
| E12 | Thr-Arg | 0.375 | 0.341 | **0.36** |
| F1 | Thr-Glu | 0.219 | 0.214 | **0.22** |
| F2 | Thr-Gly | 0.144 | 0.149 | **0.15** |
| F3 | Thr-Leu | 0.174 | 0.18 | **0.18** |
| F4 | Thr-Met | 0.263 | 0.276 | **0.27** |
| F5 | Thr-Pro | 0.165 | 0.171 | **0.17** |
| F6 | Trp-Ala | 0.256 | 0.251 | **0.25** |
| F7 | Trp-Arg | 0.374 | 0.386 | **0.38** |
| F8 | Trp-Asp | 0.297 | 0.276 | **0.29** |
| F9 | Trp-Glu | 0.197 | 0.2 | **0.19** |
| F10 | Trp-Gly | 0.272 | 0.26 | **0.27** |
| F11 | Trp-Leu | 0.25 | 0.247 | **0.25** |
| F12 | Trp-Lys | 0.317 | 0.31 | **0.31** |
| G1 | Trp-Phe | 0.175 | 0.177 | **0.18** |
| G2 | Trp-Ser | 0.243 | 0.248 | **0.25** |
| G3 | Trp-Trp | 0.299 | 0.301 | **0.30** |
| G4 | Trp-Tyr | 0.211 | 0.213 | **0.21** |
| G5 | Tyr-Ala | 0.228 | 0.186 | **0.21** |
| G6 | Tyr-Gln | 0.197 | 0.2 | **0.19** |
| G7 | Tyr-Glu | 0.196 | 0.195 | **0.19** |
| G8 | Tyr-Gly | 0.259 | 0.257 | **0.26** |
| G9 | Tyr-His | 0.221 | 0.222 | **0.22** |
| G10 | Tyr-Leu | 0.238 | 0.233 | **0.24** |
| G11 | Tyr-Lys | 0.265 | 0.258 | **0.26** |
| G12 | Tyr-Phe | 0.25 | 0.24 | **0.25** |
| H1 | Tyr-Trp | 0.262 | 0.252 | **0.26** |
| H2 | Tyr-Tyr | 0.252 | 0.248 | **0.25** |
| H3 | Val-Arg | 0.203 | 0.19 | **0.19** |
| H4 | Val-Asn | 0.189 | 0.18 | **0.19** |
| H5 | Val-Asp | 0.161 | 0.145 | **0.15** |
| H6 | Val-Gly | 0.153 | 0.149 | **0.15** |
| H7 | Val-His | 0.171 | 0.162 | **0.17** |
| H8 | Val-Ile | 0.157 | 0.147 | **0.15** |
| H9 | Val-Leu | 0.252 | 0.228 | **0.24** |
| H10 | Val-Tyr | 0.238 | 0.223 | **0.23** |
| H11 | Val-Val | 0.206 | 0.184 | **0.19** |
| H12 | γ-Glu-Gly | 0.284 | 0.263 | **0.27** |

Table S9. Biolog growth assay of ∆*dppA1dppA2* with phenotypic microarray plates 8 (PM8 peptide nitrogen sources). Peptides in wells with an average absorbance (590 nm) ≥ 0.2 were considered positive for utilization.

| Well | Sample | Absorbance (590) 1 | Absorbance (590) 2 | Average Absorbance |
| --- | --- | --- | --- | --- |
| A01 | Negative control | 0.159 | 0.156 | **0.16** |
| A02 | Positive control | 0.265 | 0.31 | **0.29** |
| A03 | Ala-Asp | 0.386 | 0.396 | **0.39** |
| A04 | Ala-Gln | 0.329 | 0.337 | **0.33** |
| A05 | Ala-Ile | 0.629 | 0.591 | **0.61** |
| A06 | Ala-Met | 0.629 | 0.646 | **0.64** |
| A07 | Ala-Val | 0.792 | 0.757 | **0.78** |
| A08 | Asp-Ala | 0.36 | 0.327 | **0.34** |
| A09 | Asp-Gln | 0.371 | 0.33 | **0.35** |
| A10 | Asp-Gly | 0.39 | 0.376 | **0.38** |
| A11 | Glu-Ala | 0.37 | 0.365 | **0.37** |
| A12 | Gly-Asn | 0.196 | 0.211 | **0.20** |
| B1 | Gly-Asp | 0.25 | 0.23 | **0.24** |
| B2 | Gly-Ile | 0.266 | 0.268 | **0.27** |
| B3 | His-Ala | 0.271 | 0.276 | **0.27** |
| B4 | His-Glu | 0.345 | 0.331 | **0.34** |
| B5 | His-His | 0.363 | 0.357 | **0.36** |
| B6 | Ile-Asn | 0.301 | 0.295 | **0.29** |
| B7 | Ile-Leu | 0.394 | 0.384 | **0.39** |
| B8 | Leu-Asn | 0.323 | 0.329 | **0.33** |
| B9 | Leu-His | 0.27 | 0.27 | **0.27** |
| B10 | Leu-Pro | 0.316 | 0.32 | **0.32** |
| B11 | Leu-Tyr | 0.356 | 0.343 | **0.35** |
| B12 | Lys-Asp | 0.202 | 0.186 | **0.19** |
| C1 | Lys-Gly | 0.326 | 0.32 | **0.32** |
| C2 | Lys-Met | 0.191 | 0.177 | **0.18** |
| C3 | Met-Thr | 0.257 | 0.246 | **0.25** |
| C4 | Met-Tyr | 0.699 | 0.554 | **0.63** |
| C5 | Phe-Asp | 0.291 | 0.273 | **0.28** |
| C6 | Phe-Glu | 0.316 | 0.303 | **0.31** |
| C7 | Gln-Glu | 0.433 | 0.399 | **0.42** |
| C8 | Phe-Met | 0.246 | 0.221 | **0.23** |
| C9 | Phe-Tyr | 0.231 | 0.216 | **0.22** |
| C10 | Phe-Val | 0.226 | 0.217 | **0.22** |
| C11 | Pro-Arg | 0.254 | 0.241 | **0.25** |
| C12 | Pro-Asn | 0.152 | 0.158 | **0.16** |
| D1 | Pro-Glu | 0.228 | 0.214 | **0.22** |
| D2 | Pro-Ile | 0.209 | 0.194 | **0.20** |
| D3 | Pro-Lys | 0.266 | 0.26 | **0.26** |
| D4 | Pro-Ser | 0.312 | 0.299 | **0.31** |
| D5 | Pro-Trp | 0.475 | 0.467 | **0.47** |
| D6 | Pro-Val | 0.269 | 0.267 | **0.27** |
| D7 | Ser-Asn | 0.258 | 0.253 | **0.26** |
| D8 | Ser-Asp | 0.229 | 0.219 | **0.22** |
| D9 | Ser-Gln | 0.215 | 0.206 | **0.21** |
| D10 | Ser-Glu | 0.272 | 0.26 | **0.27** |
| D11 | Thr-Asp | 0.123 | 0.123 | **0.12** |
| D12 | Thr-Gln | 0.137 | 0.14 | **0.14** |
| E1 | Thr-Phe | 0.212 | 0.213 | **0.21** |
| E2 | Thr-Ser | 0.171 | 0.153 | **0.16** |
| E3 | Trp-Val | 0.186 | 0.183 | **0.19** |
| E4 | Tyr-Ile | 0.194 | 0.192 | **0.19** |
| E5 | Tyr-Val | 0.198 | 0.187 | **0.19** |
| E6 | Val-Ala | 0.223 | 0.212 | **0.22** |
| E7 | Val-Gln | 0.252 | 0.251 | **0.25** |
| E8 | Val-Glu | 0.231 | 0.229 | **0.23** |
| E9 | Val-Lys | 0.294 | 0.296 | **0.29** |
| E10 | Val-Met | 0.204 | 0.191 | **0.19** |
| E11 | Val-Phe | 0.203 | 0.186 | **0.19** |
| E12 | Val-Pro | 0.199 | 0.159 | **0.18** |
| F1 | Val-Ser | 0.296 | 0.311 | **0.30** |
| F2 | β-Ala-Ala | 0.175 | 0.175 | **0.18** |
| F3 | β-Ala-Gly | 0.198 | 0.185 | **0.19** |
| F4 | β-Ala-His | 0.201 | 0.188 | **0.19** |
| F5 | Met-β-His | 0.174 | 0.161 | **0.17** |
| F6 | β-Ala-Phe | 0.164 | 0.156 | **0.16** |
| F7 | D-Ala-D-Ala | 0.117 | 0.115 | **0.12** |
| F8 | D-Ala-Gly | 0.19 | 0.189 | **0.19** |
| F9 | D-Ala-Leu | 0.206 | 0.207 | **0.21** |
| F10 | D-Leu-D-Leu | 0.171 | 0.17 | **0.17** |
| F11 | D-Leu-Gly | 0.114 | 0.112 | **0.11** |
| F12 | D-Leu-Tyr | 0.123 | 0.122 | **0.12** |
| G1 | γ-Glu-Gly | 0.17 | 0.178 | **0.17** |
| G2 | γ-D-Glu-Gly | 0.103 | 0.103 | **0.10** |
| G3 | Gly-D-Ala | 0.165 | 0.156 | **0.16** |
| G4 | Gly-D-Asp | 0.109 | 0.108 | **0.11** |
| G5 | Gly-D-Ser | 0.12 | 0.12 | **0.12** |
| G6 | Gly-D-Thr | 0.094 | 0.094 | **0.09** |
| G7 | Gly-D-Val | 0.084 | 0.084 | **0.08** |
| G8 | Leu-β-Ala | 0.168 | 0.154 | **0.16** |
| G9 | Leu-D-Ala | 0.154 | 0.157 | **0.16** |
| G10 | Phe-β-Ala | 0.142 | 0.136 | **0.14** |
| G11 | Ala-Ala-Ala | 0.217 | 0.207 | **0.21** |
| G12 | D-Ala-Gly-Gly | 0.206 | 0.182 | **0.19** |
| H1 | Gly-Gly-Ala | 0.439 | 0.426 | **0.43** |
| H2 | Gly-Gly-D-Leu | 0.154 | 0.158 | **0.16** |
| H3 | Gly-Gly-Gly | 0.268 | 0.269 | **0.27** |
| H4 | Gly-Gly-Ile | 0.242 | 0.218 | **0.23** |
| H5 | Gly-Gly-Leu | 0.272 | 0.241 | **0.26** |
| H6 | Gly-Gly-Phe | 0.155 | 0.136 | **0.15** |
| H7 | Val-Tyr-Val | 0.157 | 0.163 | **0.16** |
| H8 | Gly-Phe-Phe | 0.158 | 0.147 | **0.15** |
| H9 | Leu-Gly-Gly | 0.216 | 0.189 | **0.20** |
| H10 | Leu-Leu-Leu | 0.182 | 0.193 | **0.19** |
| H11 | Phe-Gly-Gly | 0.227 | 0.184 | **0.21** |
| H12 | Tyr-Gly-Gly | 0.654 | 0.643 | **0.65** |

Table S10. Bacterial strains and plasmids

| **Strain/Plasmid** | **Relevant characteristics** | **Source or Reference** |
| --- | --- | --- |
| ***E. coli*** |  |  |
| DH5α | *recA1 endA1* | (1) |
| M15/pREP4 | Km^r^; *lac* *ara gal mtlF recA uvr* | Qiagen |
| S17-1 | Sm^r^ Tp^r^; *recA endA thi hsdR* RP4-2 Tc::Mu::Tn7 | (2) |
| ***S. meliloti*** |  |  |
| RU11/001 | Sm^r^; Spontaneous streptomycin-resistant wild-type strain |  |
| RU11/828 | Δ*mcpU* | (3) |
| RU13/149 | Sm^r^ Δ*mcpS* Δ*mcpT* Δ*mcpU* Δ*mcpV* Δ*mcpW* Δ*mcpX* Δ*mcpY* Δ*mcpZ* Δ*icpA* (*che*^-^) | (4) |
|  |  |  |
| BS362 | Sm^r^, ∆*dppA1* | This work |
| BS364 | Sm^r^, ∆*dppA1*∆*dppA2* | This work |
| **Plasmids** |  |  |
| pQE30 | Ap^r^; expression vector | Qiagen |
| pQE60 | Ap^r^; expression vector | Qiagen |
| pBS373 | Ap^r^; 735-bp SphI/HindIII PCR fragment containing *mcpU*, 118 to 852 bp (aa 40 to 284) cloned into pQE30 | (5) |
| pBS390 | Ap^r^; 735-bp SphI/HindIII PCR fragment containing *mcpU*, 118 to 852 bp (aa 40 to 284), with *mcpU* codon 182 changed from GAT to GAG (D182E) cloned into pQE30 | (5) |
| pBS767 | Ap^r^; 1524-bp NcoI/BamHI PCR fragment containing *dppA1* cloned into pQE60 | This work |

Table S11. Links to 6-second time-lapse movies of bacterial responses to agarose-ligand plugs

| No ligand | https://www.mediafire.com/file/cmqtt1n02uqqk8d/No_ligand.mp4/file |
| --- | --- |
| Proline | https://www.mediafire.com/file/qocsxkakf9coi1n/proline.mp4/file |
| Trp-Ala | https://www.mediafire.com/file/cdzdytadewjawkr/Trp-ala.mp4/file |
| Ala-Ala | https://www.mediafire.com/file/b4nkolivb2yldev/Ala-ala.mp4/file |

A. B.

**Fig. S1. Isothermal titration calorimetry of McpU^LBD^ with alanine and Ala-Ala after saturation with alanine**. (A) Titration of 150 µM ala-saturated McpU^LBD^ with 0.8 mM ala, (B) Titration of ala-saturated McpU^LBD^ with 5 mM Ala-Ala.

Bacteria per capillary (×104)

| Concentration (mM) | WT | ∆dppA1 | ∆dppA1dppA2 |
| --- | --- | --- | --- |
| 0.1 | 0 | 0 | 0 |
| 1 | 1366.81 | 572 | 994 |
| 10 | 9527.78 | 6733 | 4560.67 |
| 100 | 31166.7 | 55116.7 | 33116.3 |

**Fig. S2.** Capillary chemotaxis assay of *S. meliloti* wild type and dipeptide transporter deletion strains to Ala-Ala. Error bars represent the standard deviation of the mean of three biological replicates.

A.

**B.**

**Fig. S3.** (A) Biolog growth assay of *S. meliloti* wild type and deletion strain ∆*dppA1dppA2.* (B) Comparison of dipeptide utilization between WT and ∆*dppA1dppA2* for Ala-Ala, Phe-Ala, and Trp-Ala. Each bar is representative of the number of wells with metabolically active bacteria per Biolog plate. Bacteria in wells with a measured absorbance at 590 nm of ≥ 0.2 were considered metabolically active.

**Fig. S4.** Capillary chemotaxis assay of the ∆*dppA1dppA2* strain to His-Pro and Val-Phe. Error bars represent the standard deviation of the mean of three biological replicates.

References

1. Hanahan D, Meselson M. Plasmid screening at high colony density (Recombinant DNA; bacterial colony hybridization; nitrocellulose filters; chloramphenicol amplification; colony storage). Vol. 10, Gene. 1980.
2. Simon, R., O’connell, M., Labes, M., & Pühler, A. Plasmid vectors for the genetic analysis and manipulation of *Rhizobia* and other gram-negative bacteria. *Methods in enzymology*. 1986. 118, 640-659.
3. Meier VM, Muschler P, Scharf BE. Functional analysis of nine putative chemoreceptor proteins in *Sinorhizobium meliloti*. J Bacteriol. 2007 Mar;189(5):1816–26.
4. Riepl H, Maurer T, Kalbitzer HR, Meier VM, Haslbeck M, Schmitt R, Scharf B. Interaction of CheY2 and CheY2-P with the cognate CheA kinase in the chemosensory-signalling chain of Sinorhizobium meliloti. Mol Microbiol. 2008 Sep;69(6):1373-84. doi: 10.1111/j.1365-2958.2008.06342.x. Epub 2008 Jun 28. PMID: 18573176.
5. Webb BA, Hildreth S, Helm RF, Scharf BE. *Sinorhizobium meliloti* chemoreceptor McpU mediates chemotaxis toward host plant exudates through direct proline sensing. Appl Environ Microbiol. 2014;80(11):3404–15.

1. Glutamine is a default positive control in the Biolog plates (PM 6-8) and past studies from our lab have shown that McpU^LBD^ binds to glutamine; thus, this serves as an appropriate control for our experiment.
